# Recent Advances in Suspended 2D Materials and Their Applications

**DOI:** 10.3390/nano15120929

**Published:** 2025-06-15

**Authors:** Xuanshuo Zhang, Min Li, Qingya Wang, Yuxian Liang, Jing Wei, Hongbo Li, Fangze Liu

**Affiliations:** 1 School of Interdisciplinary Science, Beijing Institute of Technology, Beijing 100081, China; xszhang@bit.edu.cn (X.Z.); 3120235313@bit.edu.cn (M.L.); 3120246370@bit.edu.cn (Q.W.); 3220232629@bit.edu.cn (Y.L.); 2 Beijing Key Laboratory of Construction-Tailorable Advanced Functional Materials and Green Applications Experimental Center of Advanced Materials, School of Materials Science and Engineering, Beijing Institute of Technology, Beijing 100081, China; weijing@bit.ed.cn (J.W.); hongbo.li@bit.edu.cn (H.L.); 3 School of Interdisciplinary Science, Beijing Institute of Technology, Zhuhai 519088, China

**Keywords:** two-dimensional materials, suspended structure, transfer methods, sensors, optoelectronic devices

## Abstract

Two-dimensional (2D) materials have attracted significant attention, owing to their atomically thin thickness; large specific surface area; and excellent mechanical, optical, and electronic properties. Suspended 2D materials, which eliminate substrate effects, exhibit unique potential in a variety of applications, including ultrasensitive sensors, flexible electronic devices, acoustic devices, and optoelectronic devices. However, a central challenge in the fabrication of high-quality suspended structures lies in transfer technology—how to accurately transfer atomically thin layers onto target substrates or form self-suspended structures without introducing contamination or causing mechanical damage. This review summarizes recent advances in the fabrication, characterization, and applications of suspended 2D materials. We focus particularly on transfer methods, offering a comparative analysis of their advantages and limitations, and conclude with insights into future directions and remaining challenges.

## 1. Introduction

Two-dimensional (2D) materials refer to crystalline materials composed of single or few atomic layers, with thicknesses at the nanoscale or even in the monolayer regime, while their lateral dimensions extend to micrometers or larger scales. Two-dimensional materials, such as graphene, black phosphorus, transition metal dichalcogenides (TMDs), hexagonal boron nitride (h-BN), and 2D hybrid perovskites, have demonstrated significant application potential in optoelectronic devices [1,2,3,4,5], flexible electronics [6,7,8], and mechanical systems [9,10,11], owing to their exceptional physical properties endowed by atomic-scale thickness [12,13,14,15]. Characterized by large specific surface areas, tunable electronic band structures, outstanding optical responses, and superior mechanical properties, these materials exhibit significantly different characteristics compared to conventional three-dimensional (3D) materials [16,17,18,19,20,21].

The atomic-level structure of 2D materials renders their intrinsic properties highly sensitive and susceptible to environmental influences. When 2D materials are integrated with substrates, interfacial interactions between the substrate and the material introduce strain, doping, and disorder, significantly compromising their performance. The electrical properties of 2D materials often degrade due to substrate-induced charge scattering [22] and unintentional doping [17,23,24], which arise from these interfacial effects. Graphene supported on SiO_2_ substrates exhibits altered local band structures due to surface impurity-induced charge scattering and surface-roughness-induced bending strain, leading to nonuniform carrier density (δn ≈ 10^12^ cm^−2^) and restricted mobility [25]. Their intrinsic optical properties become obscured by interfacial reflection and optical interference effects from the substrate, while substrate confinement induces stress concentration that may trigger structural failures such as crack propagation. Furthermore, interfacial coupling can break material symmetry and reconstruct band structures. For epitaxial graphene on SiC substrates, interactions between the buffer layer and the substrate induce A/B sublattice symmetry breaking, opening a bandgap of ~0.26 eV. However, as the layer count increases beyond four, weakened substrate effects drive the bandgap toward closure [22]. The scattering effects and high interfacial thermal resistance at the substrate–2D material interface induce significant degradation in thermal conductivity. The in-plane thermal conductivity exhibits a decreasing trend with material thickness reduction, whereas out-of-plane heat transport is more prominently constrained by substrate-induced effects. Furthermore, interfacial coupling mechanisms may amplify the intrinsic anisotropy of materials, resulting in particularly pronounced substrate-imposed limitations on out-of-plane thermal conduction [26,27].

Suspended 2D material systems have gained extensive attention since the substrate effect can be eliminated, thereby enabling intrinsic property characterization free from substrate-induced doping/scattering, providing more degrees of freedom in device design and improving device performance [12]. Experimental data obtained through atomic force microscopy (AFM) nanoindentation techniques demonstrate that freestanding monolayer graphene exhibits exceptional mechanical properties: an elastic modulus as high as 1.0 TPa and intrinsic strength reaching 130 GPa, values approaching the theoretical limits of graphene [18]. Its maximum tensile strain is about 20% [28]. Functionally speaking, suspended graphene has atomic level impermeability to gases such as helium, making it a promising ultra-thin barrier material [29,30]. Beyond graphene, other 2D materials also exhibit superior in-plane mechanical properties. For instance, monolayer h-BN demonstrates a Young’s modulus of 865 GPa, a strength of 70.5 GPa, and an exceptional fracture strain of up to 17%, while monolayer molybdenum disulfide (MoS_2_) achieves a Young’s modulus of 270 GPa, a strength of 22 GPa, and a fracture strain range of 6–11% [31]. In addition, the suspended bilayer h-BN exhibits a room-temperature thermal conductivity of 646 Wm^−1^K^−1^, surpassing bulk h-BN materials yet inferior to 751 Wm^−1^K^−1^ of monolayer h-BN [32,33].

The environmental coupling effects on 2D materials exhibit multidimensional modulation mechanisms over their structural and functional properties, governed by synergistic interfacial configurations. In fully bonded architectures, substrates interact with 2D materials via strong chemical bonding or interactions, inducing significant electronic state hybridization, lattice strain, and charge transfer. Concurrently, lattice mismatch-driven local reconstruction masks intrinsic material properties and compromises electronic transport and optical responses. In contrast, quasi-freely supported systems (e.g., graphene substrates or Sn-intercalated structures) exploit weak van der Waals coupling and interfacial screening to mitigate substrate interference. This enables large-scale growth of silicene or epitaxial graphene on defect-minimized substrates, effectively suppressing substrate-induced band structure distortion and enhancing intrinsic material performance. While suspended configurations preserve pristine material characteristics, their practical implementation faces challenges in environmental stability and fabrication difficulties. These distinct interfacial paradigms collectively underscore the critical balance between performance optimization and structural robustness in 2D material engineering [34,35,36,37].

Suspended 2D materials have demonstrated unique potential in various fields such as ultra-sensitive sensors, flexible electronic devices, acoustic devices, and optoelectronic devices. Innovative applications of suspended graphene continue to push performance boundaries through unique structural designs. Bunch et al. fabricated suspended graphene nanoelectromechanical (NEMS) resonators operating at fundamental resonance frequencies in the megahertz range, achieving room-temperature charge sensitivity of 8 × 10^−4^ electrons per Hertz. While already demonstrating exceptional performance, further enhancements in quality factor (Q) and sensitivity are attainable through process optimizations and cryogenic operation [38]. Prechtel et al. developed an ultrafast suspended self-biased graphene modulator capable of femtosecond-level optoelectronic response manipulation, where the built-in electric field at graphene-metal interfaces generates photocurrents with a full-width-half-maximum of ~4 ps, while the photothermoelectric effect induces currents exhibiting a decay time of ~130 ps [39]. Abbas et al. developed a gas sensor based on suspended black phosphorus FETs and NO_2_, achieving a sensitivity as low as 5 ppb. This work highlights the critical electronic and sensing properties of black phosphorus, demonstrating its potential for future research and applications [40]. Lee et al. fabricated BP chemical sensors by suspending BP flakes atop electrode pillars, leveraging dual-side adsorption sites to enhance surface utilization. This design effectively avoids substrate interface scattering effects and charge trap formation, achieving a 23% enhancement in responsivity along with faster response and recovery speeds [41]. Liu et al. fabricated multi-suspended MoS_2_ thin films on both flat sapphire substrates (FSS) and patterned sapphire substrates (PSS). The MoS_2_/PSS devices exhibited comprehensively superior performance in photocurrent, response speed, and stability compared to conventional MoS_2_/FSS structures, with a particularly pronounced enhancement at the 365 nm wavelength. This result highlights the unique advantage of patterned substrates in localizing and enhancing short-wavelength photon interactions [42]. These advancements also emphasize how structural innovation in suspended 2D materials systems enables unprecedented device performance across electronic and sensing platforms.

Despite the immense application potential demonstrated by suspended 2D materials across multiple fields, their practical development remains hindered by critical technical bottlenecks. During material fabrication, the transfer process frequently induces structural defects such as cracks and wrinkles, while etching or exfoliation processes leave residual impurity contaminants on the material surface. These combined factors lead to significant degradation in device performance. Current core challenges center on breakthroughs in material transfer techniques, which urgently require the development of precision technologies capable of achieving large-area uniform transfer with controlled defects. Specifically, the core scientific challenge lies in achieving contamination-free and mechanically undamaged processes for either atomic-scale precise positioning of ultrathin materials onto target substrates or construction of fully integrated self-supported suspended architectures [17,25,43,44,45,46].

In this review, we summarize recent advancements in the fabrication and applications of suspended 2D materials, highlighting their superior intrinsic properties, the critical importance of eliminating substrate effects, and their diverse implementations across multiple domains. Progress in this field will facilitate the translation of 2D materials from fundamental research to practical applications, demonstrating strategic significance in frontier areas such as quantum devices, ultrasensitive sensors, and flexible wearable technologies.

## 2. Fabrication of Suspended 2D Materials

**Figure 1:**
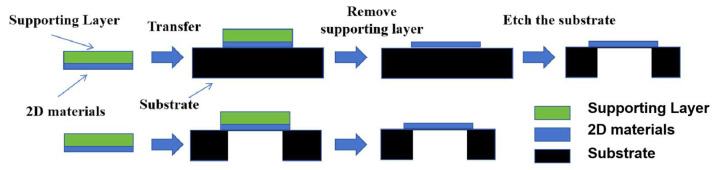
Schematic of fabrication process of suspended 2D materials. The first approach involves transferring materials onto target substrates with support layer assistance, followed by support layer removal via chemical dissolution or thermal release, coupled with optional selective substrate etching. The second strategy employs direct precision transfer of 2D materials onto pre-patterned substrates featuring micro/nano-architectures created through advanced etching processes.

The fabrication of suspended 2D materials primarily relies on two cavity construction strategies: either directly etching the substrate beneath pre-deposited or transferred 2D materials or transferring 2D materials onto pre-etched cavity structures (Figure 1). The standard fabrication process typically employs silicon dioxide substrates, beginning with oxygen plasma and ultrasonic cleaning to remove surface contaminants, followed by photolithographic patterning of the substrate combined with electron beam lithography, reactive ion etching (RIE), or chemical wet etching (using hydrofluoric acid or buffered oxide etchant) to create predefined trench structures [29]. Transfer methods for 2D materials are generally categorized into dry transfer and wet transfer techniques. After successful material transfer, minimizing subsequent processing impacts is crucial to reduce membrane damage and contamination. Post-transfer liquid drying steps often severely compromise membrane integrity due to capillary forces, a challenge effectively addressed by critical point drying (CPD) technology—this method eliminates liquid–gas interfaces through supercritical fluid phase transitions, thereby minimizing capillary stress and structural damage from membrane stretching to achieve large-area high-quality 2D films. CPD is typically employed in conjunction with wet transfer methods [47].

High-quality suspended 2D materials must satisfy rigorous structural criteria: high coverage, wrinkle-free surfaces with root-mean-square (RMS) roughness below 1 nm; uniform thickness with layer consistency; and controlled local lattice strain alongside minimized surface contaminants. For defect management, the material must maintain a defect density below 10^11^ cm^−2^ to ensure structural integrity. Additionally, these systems exhibit low absolute Raman intensity coupled with a relatively reduced signal-to-noise ratio. Currently, the field still lacks an efficient and reproducible fabrication method for high-quality suspended 2D materials, with major challenges including achieving stable, damage-free, and controllable suspensions while eliminating substrate support and maintaining 2D material integrity. Etching or stripping processes may introduce defects and contamination, leading to material inhomogeneity and unintended doping. Subsequently, the underlying mechanisms and operational protocols of dry transfer and wet transfer methodologies are elaborated in detail, with particular emphasis on their respective advantages and limitations. The detailed comparisons are summarized in Table 1.

### 2.1. Wet Transfer Methods

Based on transfer mechanisms, wet transfer methods can be classified into two categories: supported-layer and support-free transfers [54]. The wet transfer method typically relies on support layers to prevent mechanical damage. Although partial removal of these layers can be achieved through solvent rinsing or annealing, the complete elimination of residual contaminants remains a critical challenge. These residues not only degrade material performance by increasing electrical resistance [55] but also induce carrier mobility reduction and Fermi level shifts through unintended doping, while simultaneously diminishing optical transmittance [56] and overall material quality. In multilayer stacked systems, interfacial contaminant accumulation generates nanobubbles [57], increasing surface roughness that suppresses phonon transport and reduces thermal conductivity [58]. The cumulative effect of layer-by-layer transfer processes exponentially exacerbates these issues. Such contamination issues fundamentally constrain practical applications of 2D materials and obstruct accurate characterizations of their intrinsic properties [55,59,60,61,62,63].

#### 2.1.1. Wet Transfer Methods with Supporting Layer

The design and selection of support layers are pivotal for achieving efficient and high-quality transfers. An ideal support layer must satisfy three critical characteristics: (1) maintaining strong interfacial adhesion with the 2D material during transfer; (2) exhibiting high stability throughout the entire process; (3) enabling easy removal under mild conditions after transferring the 2D material to the target substrate. Poly(methyl methacrylate) (PMMA) currently dominates as the most widely used support material, while researchers have systematically explored alternative materials including polymers, organic molecules, and inorganic compounds. Although most polymer-based and metallic support layers demonstrate excellent adhesion and processing stability, they struggle with residue removal challenges. Low-molecular-weight organic molecules, despite their clean removability due to weak interfacial bonding [64], often fail to provide sufficient mechanical support during transfer. Some materials exhibit both high removability and adhesion but face limitations in standard fabrication protocols owing to their environmental sensitivity.

##### Wet Transfer Methods via Polymer

**Figure 2:**
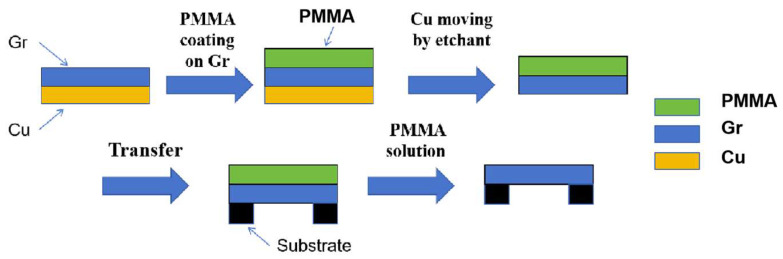
Schematic of fabrication process of suspended graphene. The process initiates with spin-coating a PMMA support layer onto graphene, followed by chemical etching of the copper substrate using FeCl_3_ solution to form a PMMA/graphene composite film. Subsequently, this composite film is precisely transferred onto target substrates (e.g., silicon wafers or flexible substrates). Finally, the PMMA support layer is completely removed through acetone dissolution or thermal annealing processes.

In wet transfer processes using polymer support layers, PMMA is commonly employed as a temporary carrier for 2D material transfer. The standard procedure involves chemically etching away the copper substrate to adhere the PMMA-supported 2D film onto a target substrate (e.g., porous-structured substrates), followed by PMMA removal via acetone dissolution (Figure 2) [49,65,66,67,68,69]. Kim et al. utilized PMMA as a support layer to transfer chemical vapor deposition (CVD)-grown graphene for fabricating suspended graphene structures, a process that effectively preserves the material’s intrinsic properties by minimizing substrate-induced interference [66].

**Figure 3:**
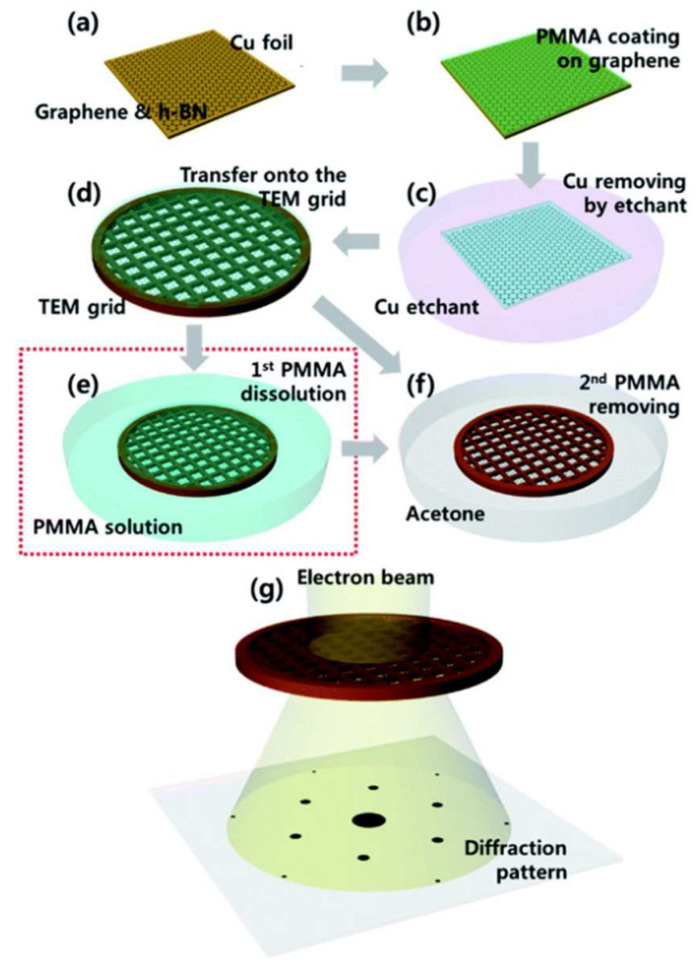
Two-step method for soft dissolving PMMA to prepare the TEM samples. (**a**) Graphene and h-BN grown on the Cu foil; (**b**) PMMA coating for supporting the layer of graphene and h-BN; (**c**) etching the Cu foil by Cu etchant; (**d**) transferring PMMA coated graphene onto the TEM grid and drying for 1 day; (**e**) first step for soft dissolving PMMA by PMMA solution; (**f**) cleaning and removal of PMMA residue by acetone as a second step; (**g**) analyzing the fabricated samples by TEM [48]. Copyright 2016, RSC adv.

In conventional fabrication processes, the rigid spin-coated PMMA layer must be completely removed with acetone after metal catalyst etching, but aggressive solvent action subjects the 2D material to high capillary stresses, causing wrinkles or fractures in large suspended areas unless handled with extreme care or assisted by specialized equipment like critical point dryers. To address PMMA support layer removal challenges in suspended 2D material preparation, Han et al. developed a two-step dissolution method that effectively resolves film collapse caused by capillary forces. First, a diluted PMMA solution (2, 4, and 9% PMMA anisole solutions) gently dissolves the coated PMMA layer on graphene for 10 min. Second, acetone removes residual PMMA for another 10 min (Figure 3) [48]. The rigid PMMA layer dissolves slowly and uniformly in the liquid PMMA solution, allowing the underlying graphene to mechanically relax and achieve better conformal contact with TEM grids. Moreover, the soft PMMA dissolution process reduces liquid-induced capillary forces compared to conventional methods. Crucially, higher-concentration PMMA solutions better preserve suspended membranes on grids than lower-concentration counterparts. This novel technique enables graphene membranes to uniformly suspend across TEM grids without tears or folds, achieving atomic-resolution HRTEM imaging while demonstrating improved process reliability and scalability for suspended 2D material fabrication.

However, this method suffers from a critical limitation: surface tension fluctuations caused by acetone evaporation induce microcracks in suspended films, particularly detrimental to suspended structure fabrication. To address this, CPD has been implemented as an optimized approach. The modified protocol first substitutes acetone with ethanol for solvent exchange, then introduces liquid CO_2_ for gradient solvent replacement. By precisely regulating pressure and temperature to reach the CO_2_ critical point, the system achieves a phase transition from liquid to supercritical fluid. Under this supercritical state, the elimination of liquid–gas interfacial effects allows gradual CO_2_ venting from the chamber, effectively circumventing structural damage from capillary forces and film stretching inherent in conventional drying. This technique significantly enhances the yield and structural integrity of large-area suspended 2D membranes [47].

**Figure 4:**
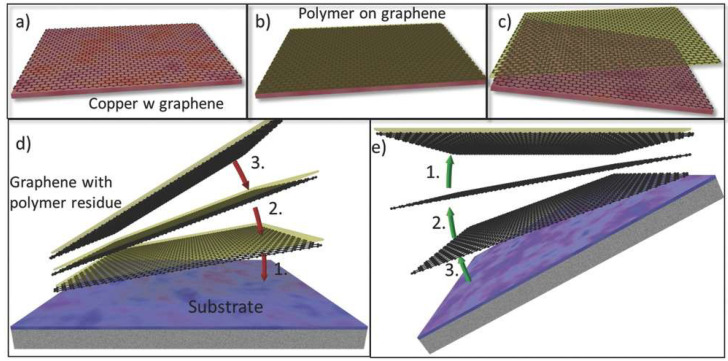
Graphene stack production: (**a**) Graphene is grown on copper by CVD and (**b**) coated by polymer for transfer. (**c**) After dissolving the copper, the graphene is transferred onto another piece of graphene on copper, and the process is repeated until the desired number of layers has been transferred. (**d**) The conventional transfer with polymer residue between layers works by building the stack layer-by-layer on the final substrate. (**e**) The modified transfer without polymer residue between layers works by building the stack under one polymer layer and by transferring to the final substrate at the end [74]. Copyright 2015, Adv. Mater. Interfaces.

In addition to using CPD method to reduce the surface tension of acetone volatilization, the stability of suspended films can be further improved through the fabrication of bilayer 2D material structures (Figure 4) [70,71,72,73,74]. Monolayer graphene is precisely stacked onto a larger copper-supported monolayer graphene substrate using PMMA as a transfer medium to construct a bilayer composite structure. The entire bilayer system is then transferred to the target substrate via standard wet transfer protocols [75]. Compared to single-layer configurations, the bilayer architecture significantly reduces localized stress nonuniformity through interlayer stress redistribution, thereby effectively mitigating structural damage caused by excessive surface tension during acetone evaporation. This approach improves the success rate of large-area transfer processes [76,77]. Fan et al. demonstrated that suspended bilayer graphene ribbons integrated with silicon proof masses can function as combined spring-mass and piezoresistive sensing systems. Fabricated using processes compatible with large-scale semiconductor manufacturing techniques, these sensors achieve a device footprint at least two orders of magnitude smaller than conventional state-of-the-art silicon accelerometers while maintaining high sensitivity [76].

**Figure 5:**
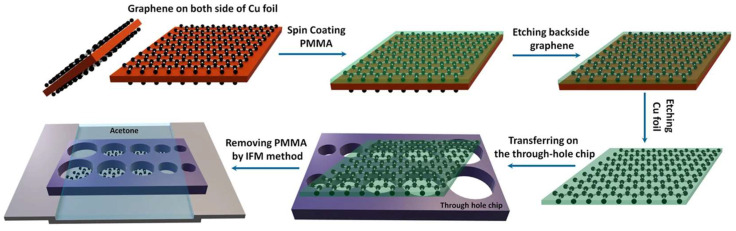
CVD graphene transferring process. The schematic of the transferring procedure for fabricating large suspended graphene membranes. Suspended CVD graphene was fabricated using typical procedures, including PMMA spin-coating, backside graphene etching, copper foil etching, rinsing, and wet-transfer onto a perforated SiO_2_/Si substrate. After the PMMA/graphene membrane was thoroughly dried, PMMA was removed using the Inverted Floating Method [79]. Copyright 2020, Sci. Rep.

Furthermore, the inverted floating method (IFM) can be employed to optimize 2D material transfer processes. This approach involves suspending the PMMA-2D material composite structure upside-down above an acetone solution, ensuring the 2D film avoids direct contact with liquid acetone. This configuration enables unidirectional downward dissolution of the PMMA layer, effectively preventing acetone infiltration from structurally compromising the 2D material. Following complete PMMA dissolution, a gradient solvent replacement strategy is implemented using low-surface-tension methoxynonafluorobutane (C_4_F_9_OCH_3_) to gradually displace residual acetone. The introduction of this fluorinated solvent significantly reduces capillary stresses exerted on the film during solvent evaporation. Finally, the 2D material membrane undergoes thorough cleaning. By isolating the 2D film from acetone exposure, the IFM method enhances the success rate of large-area suspended 2D membrane transfers while minimizing interfacial damage [50,78,79]. Akbari et al. successfully fabricated ultraclean monolayer and bilayer CVD graphene membranes with diameters up to 500 μm and 750 μm, respectively, using a suspended inverted fabrication approach (Figure 5) [79]. Meanwhile, Wan et al. designed a novel large-area suspended graphene release device based on the IFM through simulation-driven analysis of dynamic processes in traditional graphene suspension release. This optimized technique achieved a near 50% success rate for transferring 200 μm-diameter monolayer suspended graphene onto stainless steel substrates, representing a significant improvement over conventional immersion-based methods [50].

Additionally, poly(bisphenol A carbonate) (PC) can serve as an alternative support layer for the wet transfer processes. The 2D material is first transferred to the target substrate using PC as the carrier, followed by PC removal using chloroform or acetone, complemented by CPD for final cleaning. However, the rapid dissolution of PC in pure chloroform may severely damage or tear the 2D material. To mitigate this, a gradual solvent transition strategy is implemented by buffering chloroform with varying concentrations of acetone, effectively preventing 2D materials damage during dissolution. Compared to PMMA, PC demonstrates reduced polymeric residue after removal. The Raman spectra of PC-transferred Gr exhibit approximately 3–5 times higher noise levels compared to PMMA-coated samples, with no detectable PMMA characteristic peaks or low-frequency background signals. In PC-transferred Gr, the Raman G-band (~1584 cm^−1^) shows lower intensity but higher signal-to-noise ratio relative to PMMA-transferred Gr, while the D-band (~1324 cm^−1^) demonstrates weaker intensity. This optimized protocol combines controlled solvent gradients with CPD to minimize mechanical stress and preserve the structural integrity of transferred 2D materials [80,81].

In the evolution of 2D material transfer technologies, alternative polymer support layers such as polyvinyl alcohol (PVA), polyphthalaldehyde (PPA), hydrophobic polystyrene (PS), and paraffin have demonstrated potential alongside conventional options. However, these macromolecular materials are inherently limited by their long-chain architectures—even with rigorous cleaning protocols, nanoscale polymer fragments inevitably persist on 2D material surfaces, compromising interfacial purity and device performance. This residual contamination stems from the entanglement of high-molecular-weight polymer chains with atomic-scale defects in 2D lattices during dissolution processes. Therefore, exploring alternative materials as support layers for wet transfer methods is also a current research focus.

##### Wet Transfer Methods via Organic Molecules

In the realm of clean transfer technologies for 2D materials, organic small-molecule systems demonstrate remarkable advantages due to their unique physicochemical properties. Compared to conventional polymer support layers, these materials combine low sublimation temperatures with high solvent solubility, enabling near-complete residue-free removal. A notable example is the polycyclic aromatic hydrocarbon pentacene (C_22_H_14_) [82], which can be efficiently eliminated post-transfer through 250 °C thermal annealing or tetrahydrofuran (THF) solvent washing.

**Figure 6:**
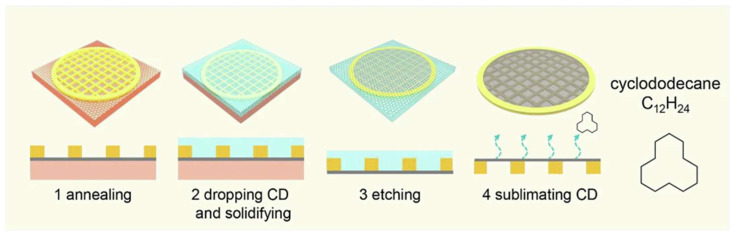
High-intactness and clean transfer method for single-layer suspended graphene with CD as a support layer. After attaching a TEM grid onto graphene assisted by evaporation of IPA, CD particles around 1 mg were introduced on graphene surface. CD was then melted through heating to fully cover the graphene surface, and it returned to a solid state to form a stable compact supporting layer at room temperature. Following the etching of the underlying copper beneath the graphene and subsequent thorough cleaning, the CD undergoes spontaneous sublimation at 40 °C [51]. Copyright 2024, Nat. Commun.

Cyclododecane (CD), a cyclic hydrocarbon, emerges as another exceptional support material [83]. This nontoxic compound exists as a solid at room temperature and exhibits a vapor pressure of 1.33 kPa at 100 °C, allowing complete sublimation under ambient atmospheric conditions without residue. Through melt-recrystallization, CD forms a crack-suppressing rigid protective layer [84]. Wang et al. leveraged this property by coating molten CD onto 2D materials (e.g., graphene or MoS_2_) after adhesion to target substrates via isopropyl alcohol (IPA) evaporation-induced surface tension. The CD support layer enabled damage-free suspended membrane fabrication through subsequent sublimation, showcasing its dual functionality as both transfer medium and structural reinforcement (Figure 6) [51].

Furthermore, anthracene [85,86], naphthalene [87], camphor [88,89,90], and other organic small molecules can serve as support layers for suspended 2D material fabrication, employing transfer methodologies analogous to CD [86]. These low-molecular-weight compounds exhibit significantly weaker interfacial adhesion to 2D materials compared to conventional polymers [64], facilitating complete removal through organic solvents for cleaner transfer outcomes.

**Figure 7:**
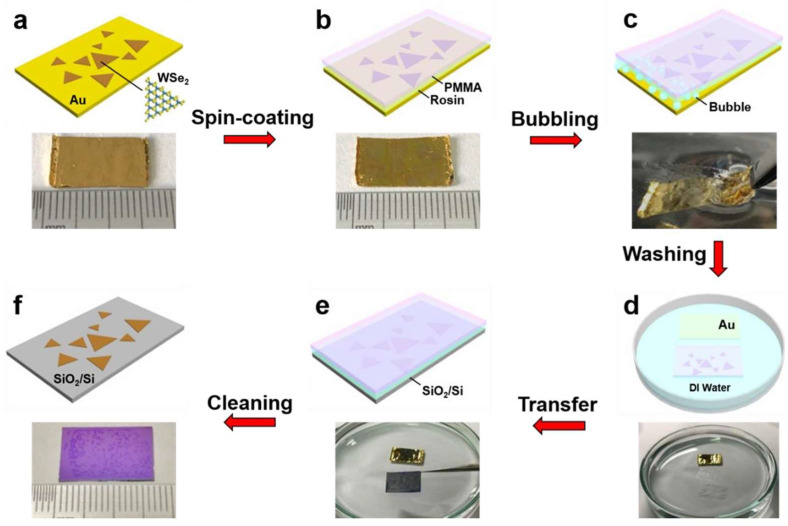
Transfer process of CVD-grown WSe_2_ on Au foil using the electrochemical bubbling method and the PMMA/rosin double support layer. (**a**) CVD-grown WSe_2_ on a Au substrate. (**b**) A PMMA/rosin/WSe_2_/Au stack obtained by spin-coating a thin layer of rosin and then PMMA. (**c**) Separation of the PMMA/rosin/WSe_2_ stack from the Au foil driven by H_2_ bubbles. (**d**) Separated PMMA/rosin/WSe_2_ stack and Au substrate in water. (**e**) The PMMA/rosin/WSe_2_/target substrate stack obtained by collecting the floating PMMA/rosin/WSe_2_ with the target substrate. (**f**) WSe_2_ on the target substrate, which was obtained after removing PMMA/rosin by repeatedly washing in warm acetone and rinsing with isopropanol, and then drying under a high-purity N_2_ flow [91]. Copyright 2019, ACS Nano.

However, while this reduced adhesion benefits residue-free transfer, it simultaneously introduces insufficient mechanical protection—fluid turbulence and surface tension during wet transfer processes often induce microcracks in 2D materials. To resolve this contradiction, a hybrid organic molecule/polymer support system has been developed. This architecture integrates organic molecules as buffer layers between 2D materials and traditional polymers (e.g., PMMA or PDMS), creating a “rigid–flexible” sandwich structure. For instance, rosin can be combined with PMMA to form a dual-support system: the organic molecular layer provides stress buffering and residue-free removal capabilities, while the polymer layer ensures mechanical stability during transfer (Figure 7) [55,91].

#### 2.1.2. Wet Transfer Methods Without Supporting Layer

To achieve polymer-free residue while maintaining structural integrity in 2D material transfers, alternative approaches bypassing conventional support layers have been developed. Although eliminating support layers prevents contamination risks, unsupported 2D membranes become vulnerable to environmental disturbances and capillary forces during wet transfer, leading to frequent rupture. To balance residue-free requirements with high structural fidelity, two strategies have emerged: frame-assisted transfer and substrate supported transfer.

##### Frame-Assisted Transfer

Mechanical frameworks typically perform microfabrication on silicon or polymer grids to stabilize 2D materials during solution processing, while achieving residue free separation after transfer through selective etching [92,93,94].

**Figure 8:**
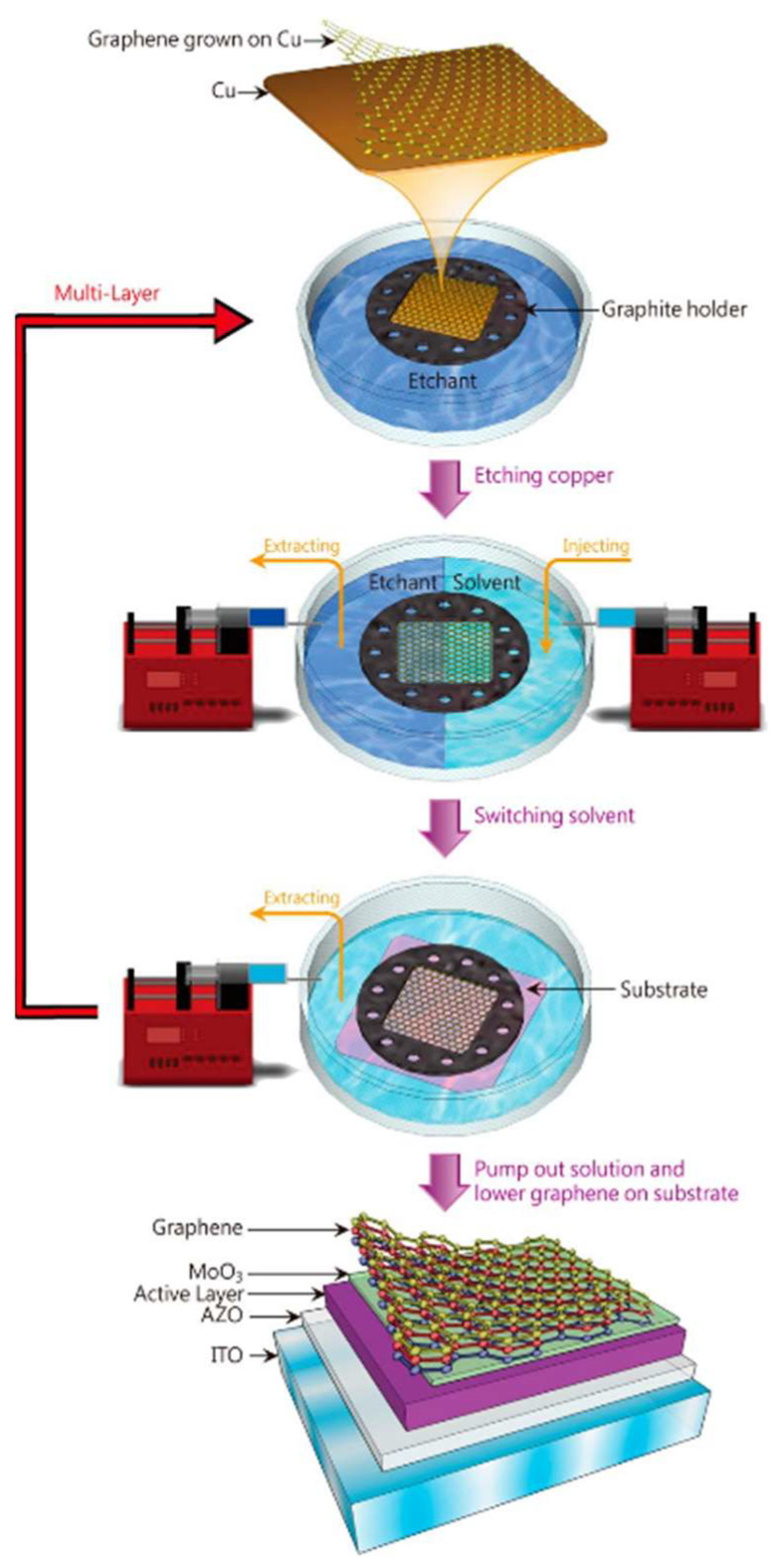
Schematic illustration of the polymer-free transfer process. The process initiates by positioning the graphene scaffold at the liquid–gas interface of an etchant comprising IPA and 0.1 M ammonium persulfate (APS), leveraging spatial confinement to stabilize monolayer graphene floating at the interface post-copper etching. Subsequently, a dual-syringe system enables synchronized pumping-out of the etchant (0.3 mL/min) and infusion of water/IPA mixture, achieving surface tension-regulated gradient solvent replacement. Upon complete etchant substitution, the target substrate is aligned beneath the floating graphene, followed by uniform solution withdrawal to realize damage-free film-substrate lamination. Finally, thermal annealing is conducted at 60 °C for 10 min under nitrogen atmosphere [59]. Copyright 2014, ACS Nano.

Lin et al. proposed a polymer-free graphene transfer technique by replacing conventional polymer supports with microstructured graphite frameworks for mechanical protection [59]. First, graphite micropillar array was constructed as support structures on copper foil surfaces. A dynamic fluid replacement strategy is then implemented: while pumping out etchant solutions (e.g., ammonium persulfate), a water/IPA mixture is simultaneously infused at matched flow rates to maintain interfacial stability and reduce surface tension (Figure 8). This coordinated fluid exchange mechanism minimizes capillary forces during copper substrate etching while preserving graphene integrity through continuous liquid-phase support.

##### Substrate Supported Transfer

In target-substrate-supported clean transfer techniques (also termed direct transfer), the destination substrate itself functions as a temporary support structure to protect the 2D material. The core innovation lies in achieving atomic-level conformal contact between the 2D material and the substrate, enabling stable transfer through strong interfacial coupling. Regan et al. first reported this method by directly covering the TEM grid on copper supported graphene and applying IPA to wet the interface. The capillary force generated during IPA evaporation leads to seamless graphene grid adhesion [95].

**Figure 9:**
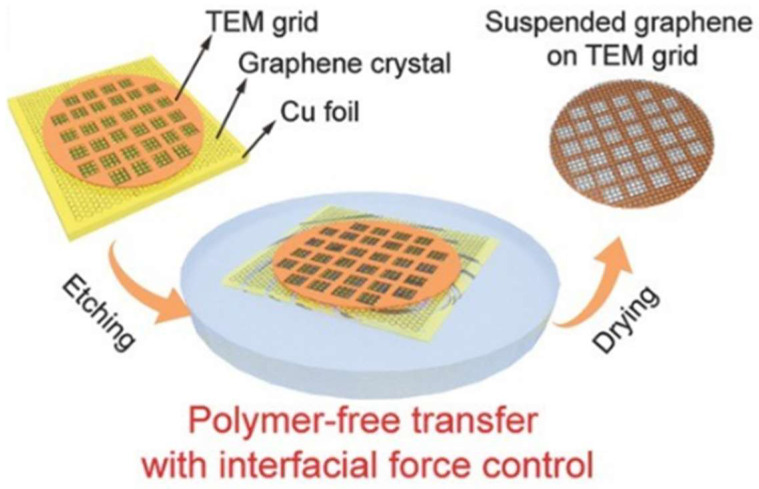
High-intactness transfer of suspended large single-crystal graphene membranes, including the steps of copper etching, graphene rinsing interfacial control by the gradual replacement of etching solution with IPA, and drying. In particular, the etching solution was gradually replaced with IPA to reduce the interfacial force during the rinsing step, which is important for the optimizing of the intactness of the graphene membrane [96]. Copyright 2017, Adv. Mater.

Zhang et al. achieved polymer-free, clean transfer of sub-centimeter-scale graphene single crystals onto TEM grids for fabricating large-area, high-quality suspended graphene membranes (Figure 9) [96]. By precisely controlling interfacial forces during the transfer process, the structural integrity of large-area graphene films reached up to 95%. Leveraging this advancement, the team further developed graphene liquid cells through π–π stacking of two pristine single-crystal graphene membranes on TEM grids. This approach eliminates polymer-induced contamination while maintaining atomic-level flatness across centimeter-scale suspended regions, enabling high-resolution TEM characterization of dynamic processes at liquid–solid interfaces.

**Figure 10:**
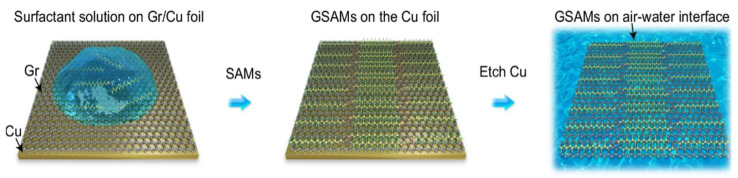
Schematic illustration showing the on-site assembly of GSAMs superstructure. After the evaporation of surfactant solution (**left**), the self-assembled monolayers of surfactant molecules are spontaneously formed on the graphene/copper surface (**middle**). The free-standing GSAMs superstructure on the water surface can be achieved after the copper foil is etched away (**right**) [52]. Copyright 2024, Nat. Commun.

Furthermore, Zheng et al. developed a robust methodology for high-resolution cryo-electron microscopy (cryo-EM) analysis by engineering a graphene–surfactant hybrid superstructure termed GSAM [52]. This method dissolves surfactant self-assembled monolayers (SAM) in IPA, and based on the aforementioned direct transfer method, combines SAM with graphene film to form a new suspended structure, creating a molecularly ordered interface while immobilizing biomolecule samples and minimizing background noise (Figure 10). This dual-functional interface addresses longstanding challenges in cryo-EM sample preparation by combining graphene’s mechanical stability with SAM-enabled biochemical precision, establishing a universal platform for next-generation structural biology investigations.

### 2.2. Dry Transfer Methods

Compared to wet transfer, dry transfer is general cleaner and simpler, making it particularly suitable for lab-scale fabrication and physical property characterization. The primary dry transfer methods are mechanical exfoliation and dry stamp transfer. Future research should prioritize developing highly accurate, precise, large-area, and efficient fabrication techniques.

#### 2.2.1. Mechanical Exfoliation

**Figure 11:**
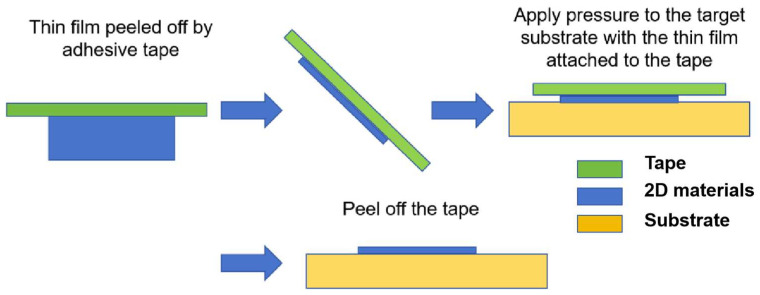
Mechanical exfoliation. Utilize adhesive tape to mechanically exfoliate the 2D material film, transfer the tape-adhered film onto the target substrate, and subsequently peel off the tape.

Mechanical exfoliation, as a classical method for preparing 2D materials, comprises three major steps: (1) repeatedly cleaving bulk crystals using adhesive tape to obtain few-layer 2D materials, (2) pressing the tape-attached flakes onto target substrates (e.g., SiO_2_/Si) for intimate contact, and (3) gently peeling off the tape after thermal treatment (typically 150–200 °C) to weaken interfacial adhesion during cooling (Figure 11) [18,38,97,98,99]. The main advantage of this approach is its complete elimination of chemical etching processes—unlike conventional metal-based substrate etching (e.g., Ni, Cu), which requires highly corrosive agents such as iron nitrate, ferric chloride, or ammonium persulfate. These chemical treatments not only incur substantial processing costs but also induce doping through residual metal ions from the etchant. By eliminating such chemical interventions, mechanical exfoliation preserves the intrinsic electronic properties of 2D materials while achieving atomically clean interfaces, making it indispensable for fundamental studies requiring pristine material characteristics. The technique’s simplicity and reproducibility have established it as a foundational methodology in nanomaterial research, particularly for prototyping quantum devices and investigating thickness-dependent physical phenomena.

Bunch et al. [29] utilized mechanical exfoliation technology to facilitate the precise fabrication of graphene micro/nanoelectromechanical devices through a three-step process: (1) pre-etching micrometer-scale trench structures on SiO_2_ substrates; (2) mechanically exfoliating graphene flakes to bridge the trenches, forming doubly clamped beams or cantilever configurations stabilized by van der Waals forces; and (3) integrating prefabricated gold electrodes within the trench gaps to establish low-resistance electrical contacts for measurement circuits. The number of graphene layers can be determined by Raman spectroscopy. The technique establishes a high-purity platform for prototyping NEMS, enabling fundamental studies of suspended graphene devices with preserved quantum characteristics and minimal interfacial contamination [10,11,43,100,101,102,103,104,105,106,107,108,109,110,111,112,113,114].

#### 2.2.2. Dry Stamp Transfer

**Figure 12:**
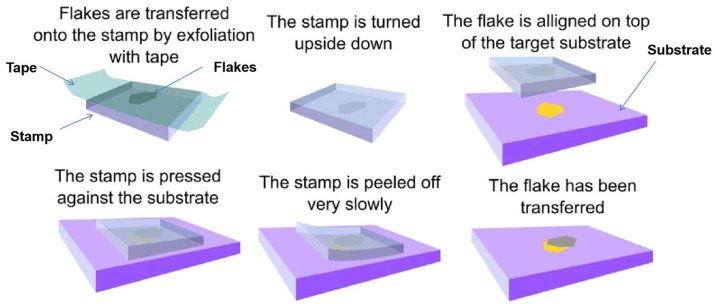
Schematic diagram of the experimental setup employed for the all-dry transfer process. (1) Flakes are transferred. (2) The stamp is turned upside down. (3) The flake is aligned on top of the stamp by exfoliation upside down of the target substrate with tape. (4) The stamp is pressed against the substrate. (5) The stamp is peeled off very slowly. (6) The flake has been transferred [119]. Copyright 2014, 2D Mater.

Dry stamping transfer technology, centered around elastomeric stamps, enables damage-free transfer of 2D materials through precise modulation of interfacial adhesion forces. This methodology exploits the strong adhesion between elastomers (e.g., PDMS) and 2D materials to delaminate flakes from their native substrates, followed by controlled release onto target substrates via superior interfacial bonding—all executed without liquid-phase intermediaries, thereby minimizing contamination risks (Figure 12) [115,116,117,118,119,120,121,122,123,124]. For example, Moreno et al. extracted graphene flakes from pyrolytic graphite using PDMS stamps in 2009 [125], and Andres et al. transferred MoS_2_ using PDMS in 2012 [53].

**Figure 13:**
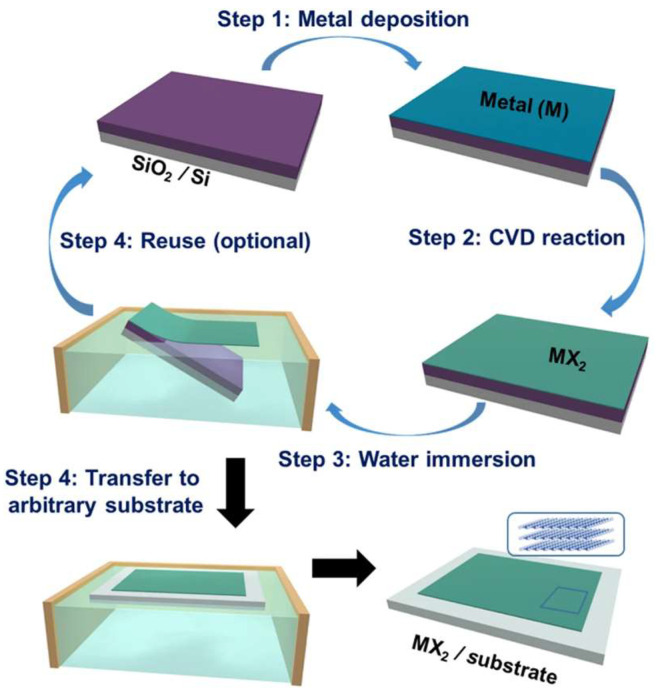
Schematic to illustrate the water-assisted green integration of CVD-grown 2D TMD layers on arbitrary substrates: (1) Deposition of transition metals on the surface of growth substrates (i.e., SiO_2_/Si) followed by their conversion to 2D TMD layers via CVD. (2) Immersion of the 2D TMDs-grown SiO_2_/Si substrates inside water followed by spontaneous 2D layer separation. (3) Transfer and integration of the delaminated 2D TMD layers onto secondary substrates inside water. (4) Recycling of the original growth substrates for additional 2D TMDs growth (optional) [126]. Copyright 2019, Sci Rep.

In addition, researchers have developed capillary assisted transfer technology to transfer hydrophobic TMD: by introducing trace amounts of deionized water between PDMS impressions and TMD surfaces (Figure 13), researchers utilize the capillary adhesion generated on the gas–liquid–solid three-phase contact line to overcome the van der Waals interaction of natural substrates [126,127,128].

## 3. Suspended 2D Materials Properties

**Figure 14:**
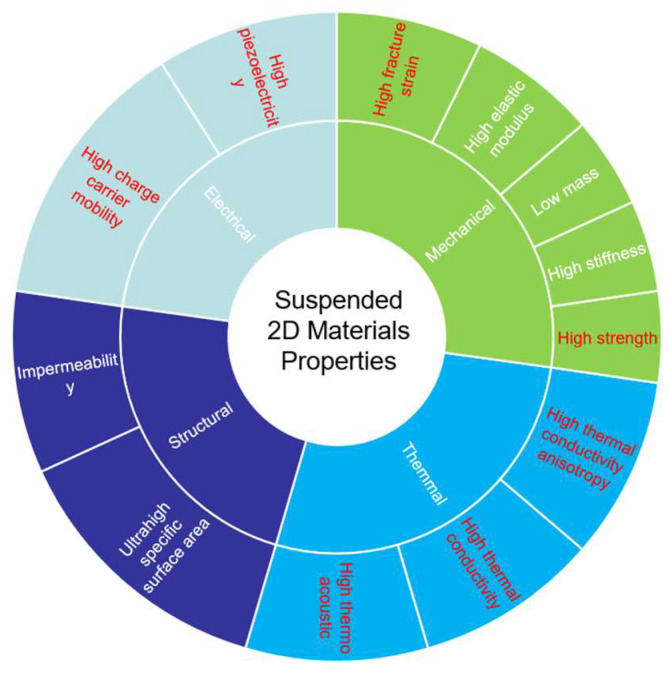
Suspended 2D materials properties. Red represents the unique properties of suspended 2D materials.

The atomic-scale thickness of 2D materials endows them with unique and exceptional optical, excitonic, mechanical, and electronic properties, demonstrating immense potential for applications in electronics, optoelectronics, and quantum technologies (Figure 14). However, this atomically thin structure renders 2D materials highly susceptible to environmental perturbations, where substrate-supported configurations often obscure intrinsic characteristics through substrate-induced strain, doping, or interfacial charge trapping. Suspended 2D materials circumvent these limitations by eliminating substrate interactions. This substrate-free architecture not only reveals fundamental material behaviors but also unlocks novel device functionalities inaccessible in supported systems.

The exceptional properties of suspended structures can be systematically characterized through multiple techniques: Optical microscopy (OM) rapidly locates suspended regions (identified by color/brightness contrast against supported areas) and provides preliminary layer count assessment. Raman spectroscopy enables layer identification, strain detection, and doping analysis through characteristic peak parameters (position, intensity, linewidth). Photoluminescence spectroscopy (PL), applicable exclusively to direct-bandgap semiconductors, determines layer thickness, band structure, and defect states. Atomic force microscopy (AFM) measures topography, step heights, surface roughness, and mechanical properties. Electrical transport characterizations require electrode fabrication, revealing intrinsic electrical performance via resistance, conductivity, and carrier mobility parameters. Thermal properties are probed by self-heated scanning thermal microscopy (detecting tip-sample heat flux), the optothermal Raman method, and micro/nano thermal bridge devices (integrating heating electrodes and temperature sensors at suspended film terminals).

When integrated with substrates, 2D materials suffer compromised electrical performance due to substrate-induced charge scattering and unintentional doping, while their intrinsic optical signatures are obscured by interfacial reflection and photon interference effects. Such substrate coupling effects fundamentally limit the exploration of intrinsic 2D material physics and necessitate suspended architectures or engineered buffer layers (e.g., h-BN encapsulation) to decouple materials from detrimental substrate interactions while preserving structural integrity and optoelectronic functionality. The integration of 2D materials with substrates often degrades their electrical performance due to substrate-induced charge scattering and unintentional doping. For instance, graphene devices supported on SiO_2_ substrates exhibit reduced carrier mobility caused by charge trap-induced scattering at the interface [25,129]. In addition, suspended 2D materials, free from defective/doped interfaces and characterized by homogeneous charge distribution, provide an ideal system for investigating quantum Hall effects [128,130,131]. Suspended graphene maintains a uniform optical absorption rate of 2.3% across a broad wavelength range without substrate-induced interference effects [132]. Shi et al. demonstrated enhanced second-harmonic generation (SHG) intensity and directional emission control by suspending monolayer WS₂ within Fabry–Pérot microcavities [133]. The scattering effects and high interfacial thermal resistance at the substrate–2D material interface induce significant degradation in thermal conductivity. The in-plane thermal conductivity exhibits a decreasing trend with material thickness reduction, whereas cross-plane heat transport is more prominently constrained by substrate-induced effects. Furthermore, interfacial coupling mechanisms may amplify the intrinsic anisotropy of materials, resulting in particularly pronounced substrate-imposed limitations on cross-plane thermal conduction [26,27]. This architecture leverages suspended 2D materials to eliminate substrate interactions. Suspended 2D materials exhibit exceptional mechanical properties, including outstanding stiffness, fracture strain, and elastic modulus, due to their large specific surface area and atomic-scale thickness, which collectively enhance structural resilience and load-bearing capacity while maintaining flexibility. The detailed comparisons are summarized in Table 2. For instance, suspended graphene demonstrates a Young’s modulus exceeding 1 TPa and intrinsic strength of ~130 GPa, approaching theoretical limits, while monolayer MoS_2_ achieves fracture strains of ~10% under uniaxial tension—performance metrics unattainable in substrate-supported configurations constrained by interfacial defects and stress gradients [18,31,134]. Additionally, the suspended bilayer h-BN exhibits a room-temperature thermal conductivity of 646 Wm⁻¹K⁻¹, exceeding that of bulk h-BN but remaining lower than monolayer h-BN [32,33].

Two-dimensional materials provide a unique platform for investigating intrinsic thermodynamic behaviors and superconducting properties. In thermal expansion studies, their atomic-scale monolayer or few-layer structures, free from substrate constraints, exhibit significantly enhanced degrees of freedom and pronounced anisotropic thermal expansion characteristics, offering critical parameters for the development of thermally responsive devices. Scattering effects and high interfacial thermal resistance at the substrate-2D material interface significantly degrade thermal conductivity. While reduced material thickness decreases in-plane thermal conductivity, cross-plane heat transport is more constrained by substrate-induced effects. Furthermore, interfacial coupling mechanisms can amplify intrinsic material anisotropy, thereby imposing more pronounced substrate limitations on cross-plane thermal conduction [26,27]. Within superconducting research, suspended architectures eliminate substrate-induced disorder effects, substantially enhancing the potential of 2D materials in superconductivity [140,141,142,143,144,145,146]. In summary, suspended 2D materials demonstrate superior electronic, mechanical, optical, and thermal properties compared to substrate-supported counterparts. The suspended configuration plays a pivotal role in unveiling intrinsic material physics, thereby advancing both fundamental explorations of low-dimensional quantum phenomena and applications in next-generation nanodevices.

## 4. Applications

Suspended 2D materials, leveraging their unique properties, are driving revolutionary advances in microelectromechanical systems (MEMS) across diverse fields—including high-sensitivity pressure sensors, micro-accelerometers, acoustic transducers, optoelectronic devices, and cryo-electron microscopy. These breakthroughs fundamentally rely on the substrate-free configuration of suspended structures. By eliminating substrate-induced scattering, loss, and charge traps, this design fully unleashes intrinsic material properties—precisely meeting the core requirements of next-generation high-performance, miniaturized, and low-power MEMS devices and enabling significant performance gains across these applications.

### 4.1. Pressure Sensor

Pressure sensors, recognized for their exceptional performance across diverse domains including gas/liquid detection, tactile sensing, and altitude measurement, are extensively deployed in industrial monitoring, environmental barometric tracking, and human–machine interfaces—applications spanning altimeters, barometers, and indoor navigation systems. The demand for high-performance pressure sensors is constantly increasing, and the current technological development presents two major trends: (1) miniaturization to reduce power consumption and shorten response time, and (2) sensitivity [21]. These advancements collectively optimize device portability, operational efficiency, and cost-effectiveness [67,147,148]. In this context, 2D materials—with their atomic thickness and ultrahigh surface-to-volume ratios—are revolutionizing high-sensitivity microsensor development by enabling strain engineering at the quantum limit and photon-matter interactions in suspended architectures [149].

Pressure sensors are broadly categorized into three types: strain-based, displacement-based, and resonant [17,150]. Strain-based variants detect pressure-induced resistance changes in 2D material membranes, delivering excellent linear response characteristics and unambiguous signal outputs. Displacement-based sensors employ optical interferometry or capacitive readouts to measure membrane deflection. And they are not easily affected by environmental factors such as gas and humidity. Each modality leverages distinct advantages of 2D material thereby expanding the operational envelope of next-generation pressure sensors across aerospace, biomedical, and IoT applications.

#### 4.1.1. Strain-Based Pressure Sensors

Piezoresistive pressure sensors (commonly referred to as strain-based pressure sensors) operate by detecting resistance variations in 2D material membranes induced by strain under pressure differentials. When a suspended 2D material membrane deflects due to applied pressure, the resulting strain alters the material’s electrical resistance, enabling pressure quantification through precise resistance monitoring. It should be noted that the gas or moisture in contact with the suspended 2D material film usually affects its resistance, thereby interfering with the piezoresistive signal during pressure measurement [151,152,153,154,155,156,157,158].

**Figure 15:**
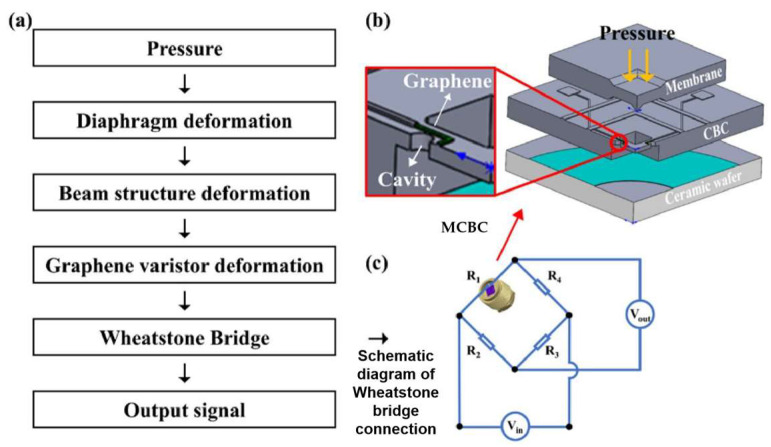
(**a**) Working principle of pressure sensor. (**b**) MCBC structure quarter model. (**c**) Schematic diagram of Wheatstone bridge connection [160]. When external pressure acts on the sensing membrane, the membrane undergoes downward deformation, mechanically driving axial displacement and bending deformation in the central bearing component (CBC). This deformation propagates to the embedded graphene piezoresistive sensor at the beam root, where lattice stretching generates tensile strain, resulting in increased electrical resistance. The resistance change disrupts the equilibrium of the Wheatstone bridge, producing a differential voltage signal with linear pressure dependence, thereby enabling high-precision quantitative measurements. Copyright 2025, ACS Appl. Electron. Mater.

Recent developments in graphene-based piezoresistive pressure sensors have achieved notable advancements, yet challenges persist in optimizing key performance metrics. Smith et al. developed a suspended monolayer graphene membrane pressure sensor with a measurement range up to 0.1 MPa, although it suffered from stability limitations [152]. Li et al. enhanced the pressure detection range to 0.13–0.18 MPa through a graphene-boron nitride heterostructure design, highlighting the potential of hybrid material systems while underscoring the need for further optimization in balancing sensitivity, stability, and operational range for practical applications [159]. Li et al. developed a wide-range graphene piezoresistive pressure sensor employing a membrane and cavity-backed beam composite (MCBC) structure to balance the trade-off between sensitivity and natural frequency. This design demonstrated a high natural frequency of 0.74 MHz across a pressure range of 0–25 MPa, while achieving localized strain of up to 0.68% in the graphene sensing resistor (Figure 15) [160].

#### 4.1.2. Displacement-Based Pressure Sensors

**Figure 16:**
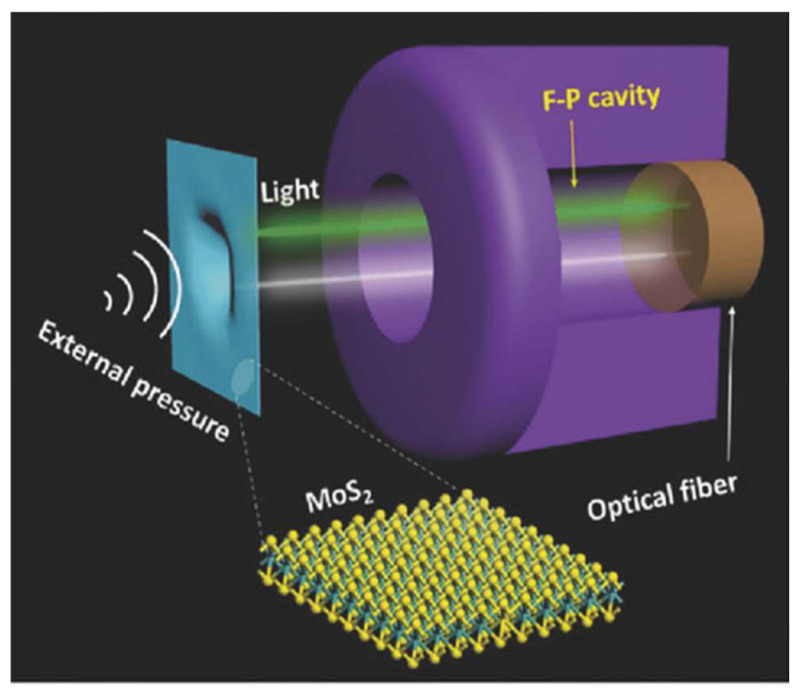
Sensor schematic diagram [163]. Laser light is coupled via optical fiber into a Fabry–Pérot (F-P) interferometric cavity, where a photodetector continuously monitors dynamic cavity length variations through acquisition of interference spectra. Phase demodulation algorithms resolve micro-displacements in cavity length, establishing a pressure-to-cavity length correlation that enables high-sensitivity detection of external pressure. Copyright 2017, Adv. Mater.

Displacement-based pressure sensors demonstrate high environmental robustness and broad applicability across automotive, aerospace, and biomedical sectors such as blood pressure monitoring, and are categorized into two primary types based on transduction mechanisms: fiber-optic and capacitive sensors. Fiber-optic variants employ laser interferometry to detect displacement at the membrane center, typically utilizing circular suspended membranes to maximize deflection amplitude under pressure gradients [161]. Ma et al. constructed a pressure sensor using few-layer graphene diaphragms with a width of 25 μm [162]. Yu et al. fabricated a miniaturized fiber-optic pressure sensor employing suspended MoS_2_ films as sensing elements, where a 125 nm-thick MoS_2_ membrane spanning an 8 μm-diameter microcavity achieved a sensitivity of 89.3 nm·Pa^−1^ with excellent linearity (Figure 16) [163].

**Figure 17:**
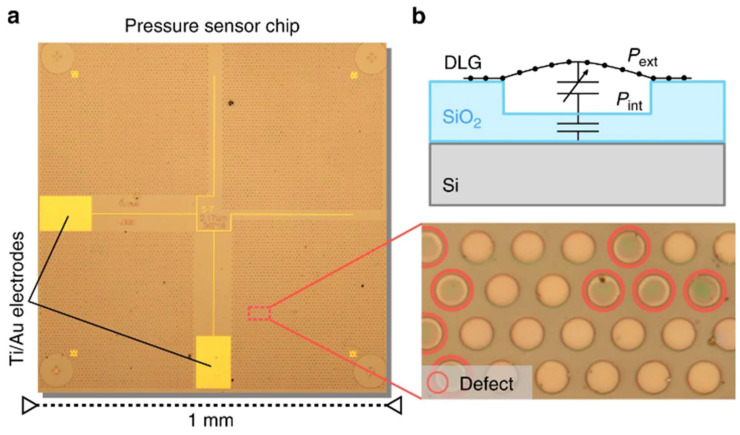
(**a**) Optical image of the sensor chip with 10,000 circular holes, a DLG/PMMA membrane, and Ti/Au electrodes. The close-up image shows the difference in contrast between intact and defect drums, with red circles indicating collapsed membranes. (**b**) Schematic device cross-section and capacitive pressure readout principle. By strictly confining the etch depth of the cavity within the thickness of the SiO_2_ layer, the design effectively prevents physical contact between graphene and the underlying silicon electrode during sensing membrane collapse, thereby fundamentally eliminating the risk of electrode short-circuiting [165]. Copyright 2020, Microsyst. Nanoeng.

Conversely, capacitive pressure sensors measure total membrane deflection through capacitance changes between the suspended membrane and a fixed bottom electrode, offering advantages including ultra-low power consumption and high sensitivity. The sensitivity is governed by parameters such as gap size, membrane thickness, Young’s modulus, pre-tension, membrane radius, and quantum capacitance in 2D materials. Notably, reducing the gap size enhances capacitive sensitivity, with maximum sensitivity occurring at zero pressure differential due to nonlinear electrostatic interactions [164]. In the realm of capacitive graphene pressure sensors, device innovations continue to drive performance breakthroughs. Davidovikj et al. achieved capacitive detection of 5 μm-diameter suspended monolayer graphene membranes by fabricating patterned bottom electrode structures on insulating substrates using micro-nanofabrication techniques [164]. In parallel, Šiškins et al. implemented a scalable integration strategy by deploying arrays of nearly 10,000 bilayer graphene membrane units within a single device (Figure 17). Each 5 μm-diameter sensing element contributed to a record areal capacitance sensitivity of 47.8 aF·Pa^−1^·mm^−2^, demonstrating how architectural scaling and collective signal amplification can transcend the limitations of individual sensor units [165].

#### 4.1.3. Resonant Pressure Sensors

**Figure 18:**
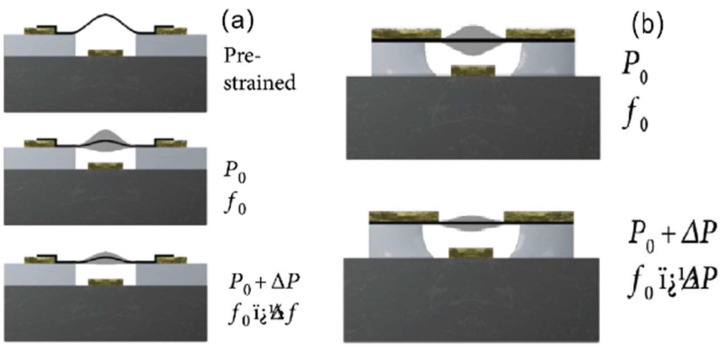
(**a**) Working principle: the gas pressure difference across the membrane causes a membrane deflection and tension change that is measured via the resonance frequency. (**b**) Working principle: the stiffness and compressibility of the gas under the membrane increases the stiffness of the membrane that is measured via the mechanical resonance frequency [166]. Copyright 2020, Research.

Resonant pressure sensors detect pressure variations through dynamic mechanical responses, primarily utilizing tension-mediated resonance or squeezed-film effects for rapid dynamic measurements (Figure 18) [166]. These resonators are categorized into resonant-tension-based pressure sensors and squeezed-film pressure sensors. Resonant-tension-based sensors operate similarly to piezoresistive variants by monitoring gas-pressure-induced strain effects on membranes. When pressure acts on a suspended membrane, the resultant stress variation shifts its resonant frequency, which is tracked via laser Doppler vibrometers for dynamic pressure monitoring [29,121,167]. Squeezed-film pressure sensors, in contrast, transcend conventional hermetic cavity constraints by exploiting gas compressibility in sub-membrane microcavities. Their operating principle relies on gas compression within the cavity when external pressure changes occur faster than the gas diffusion equilibration time, effectively “trapping” compressed gas to induce membrane deflection with a nonlinear dependence on pressure [168]. This mechanism enables high-bandwidth detection of transient pressure fluctuations, making these sensors particularly suited for chemical process monitoring and micro-drone barometric systems requiring rapid response to aerodynamic perturbations.

**Figure 19:**
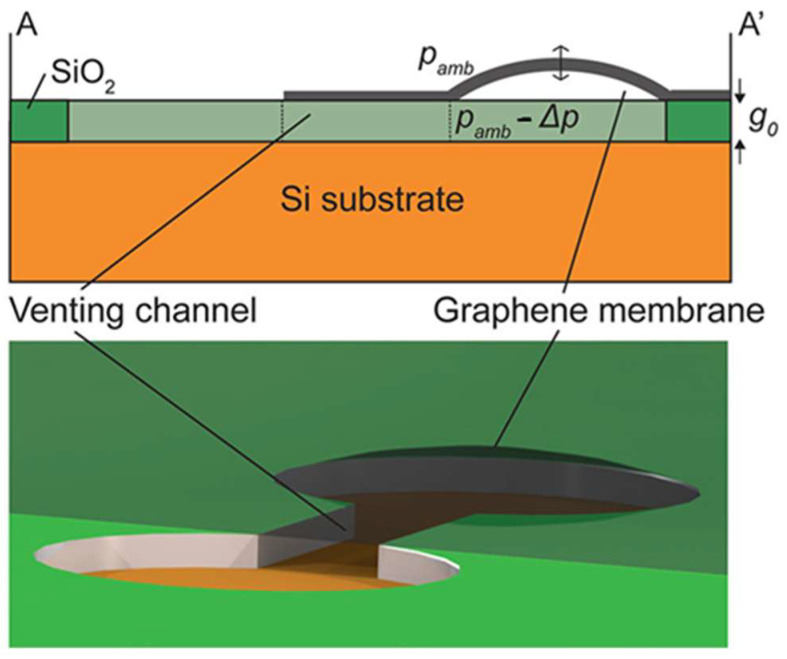
Schematic of the graphene squeeze-film sensor. This enables precise positioning of the flake to overlap half of the dumbbell configuration, thereby forming a graphene-integrated squeeze-film pressure sensor with an integrated lateral venting channel. [173]. Copyright 2016, Nano Lett.

Two-dimensional materials, leveraging their ultralow mass and exceptional mechanical responsiveness, have emerged as ideal platforms for ultrahigh-sensitivity mass detection [169,170]. The sensitivity of suspended graphene resonant mass sensors is significantly better than sensors based on silicon films, due to the low mass and high mechanical response characteristics of graphene [171]. Lee et al. demonstrated through computational analysis that graphene-based mass sensors can achieve extraordinary sensitivity levels of up to 10^−27^ g·Hz^−1^ [172]. Dolleman et al. transferred few-layer graphene onto dumbbell-shaped apertures to create an unconventional pressure sensor incorporating ventilation channels, which enabled gas pressure detection by correlating membrane deflection with pressure differentials (Figure 19) [173]. In addition, Dolleman et al. analyzed impurity concentrations on graphene surfaces by monitoring resonant frequency shifts of suspended graphene membranes during oxygen plasma cleaning [174]. These sensors have transformative potential in trace gas detection and single molecule analysis but face key challenges including environmental vibration noise and thermomechanical fluctuations. The continuous development of quality sensors is paving the way for field deployable 2D material quality sensors in environmental monitoring, precision medicine, and quantum metrology.

### 4.2. Accelerometers

In the field of MEMS accelerometers, traditional silicon-based devices face a critical size-expansion dilemma due to the conflicting requirements of maintaining a low spring constant (via long, compliant springs) and achieving large capacitive sensing areas (through densely packed interdigitated electrodes). While 2D materials offer miniaturization potential with their atomic thickness and high Young’s modulus, their intrinsically low mass limits inertial response sensitivity.

**Figure 20:**
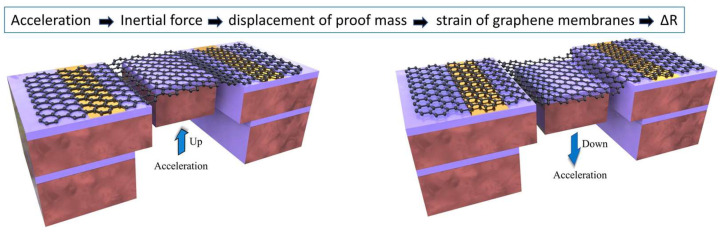
Schematic of the working principle of the graphene membranes with an attached SiO_2_/Si proof mass for sensing acceleration. At the 160 Hz excitation frequency, resonant modes of externally mounted components induce pronounced amplification of oscillation amplitudes in SiO_2_/Si proof-mass anchored to its graphene membranes. Consequently, the proof-mass displacement escalated substantially, thereby inducing critical strain gradients across the graphene diaphragms [176]. Copyright 2025, ACS Nano.

To resolve this trade-off, Fan et al. engineered a suspended bilayer graphene ribbon structure integrated with silicon proof masses using dry etching and vapor-phase HF etching [76]. Under acceleration, the graphene ribbons undergo tensile strain, enabling signal detection via their piezoresistive effect. This approach reduces accelerometer footprints by at least two orders of magnitude compared to conventional silicon counterparts. Further optimization pathways include enlarging silicon proof masses to enhance inertial effects, narrowing graphene ribbon widths, and minimizing intrinsic stress [175]. Ding et al. developed an accelerometer based on a suspended double-layer graphene membrane integrated with a silicon proof mass (Figure 20). This device achieves a mass volume reduction of at least three orders of magnitude compared to state-of-the-art silicon-based counterparts, while simultaneously enhancing sensitivity per unit volume by over three orders of magnitude. In this innovative design, the silicon mass exceeds the mass of the bilayer graphene spring by more than 30,000 times, significantly amplifying inertial response and enhancing detection capability [176]. Concurrently, the atomic-scale thinness of graphene enables efficient conversion of localized mechanical strain into measurable resistance variations, dramatically optimizing signal sensitivity. Such exceptional advantages in ultra-miniaturization and ultra-sensitive detection highlight graphene NEMSs’ application potential across multiple domains including biomedical implantable sensors, micro-nano surgical robots, and high-precision wearable devices. This technological breakthrough provides an innovative solution for next generation miniaturized intelligent systems, demonstrating graphene’s transformative capabilities in advanced microsystem engineering.

### 4.3. Acoustic Transducers in Audio Frequency

Acoustic transducers, serving as core components for electroacoustic conversion in devices like speakers and microphones, are undergoing transformative performance enhancements through the integration of 2D materials. Traditional silicon-based systems face limitations in thickness reduction and mechanical flexibility, struggling to meet the demands of wearable electronics (e.g., smart earbuds, electronic skins) for ultrathin form factors, conformal adaptability, and energy-efficient operation. Two-dimensional materials—with their atomic-scale thickness, exceptional mechanical modulus, and tunable electron–phonon coupling—offer revolutionary pathways for acoustic device innovation. Suspended 2D membranes enable unprecedented performance metrics; however, challenges persist in scalable integration processes, environmental stability under humidity/temperature cycling, and the absence of unified multiphysics models addressing coupled electromechanical–thermal–acoustic interactions [177,178].

#### 4.3.1. Speakers

**Figure 21:**
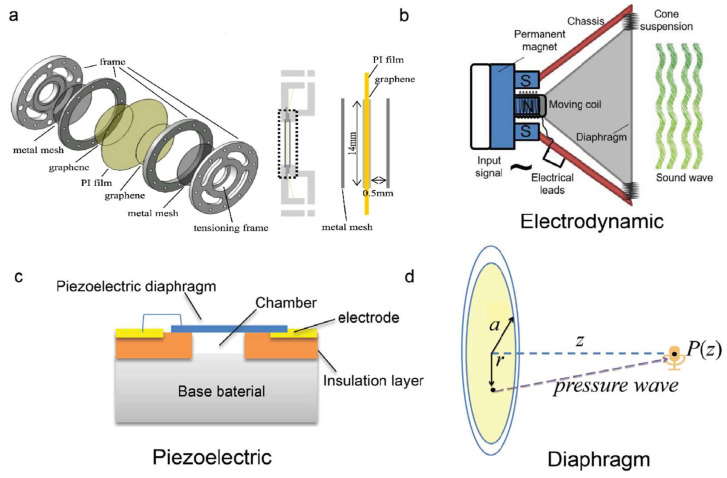
Various types of sound detection devices. (**a**) Magnified diagram of a electrostatic sound generator (**left**) and structure of the system (**right**) [179]. Copyright 2018, Nanotechnology. (**b**) Condenser microphone. (**c**) Piezoelectric microphone, generating transient current through piezoelectric effect. (**d**) Piezoresistive microphone, inducing changing resistivity through piezoresistive effect [150]. Copyright 2024, Adv. Funct. Mater.

There are four main types of speakers: electrostatic, electrodynamic, piezoelectric, and thermoacoustic (Figure 21).

##### Electrostatic Speakers

**Figure 22:**
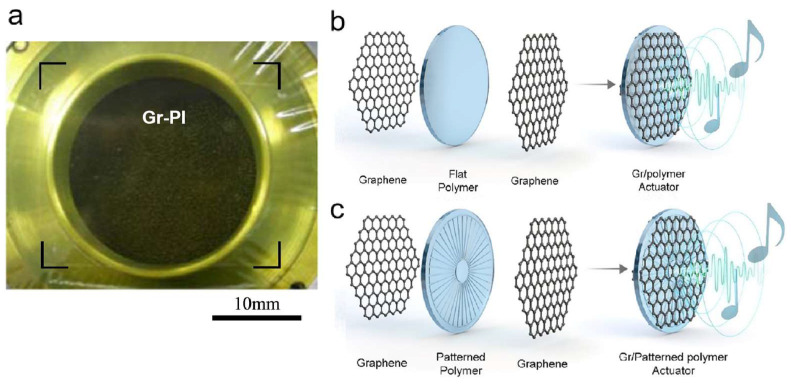
(**a**) Structure of graphene/PI diaphragm [179]. Copyright 2018, Nanotechnology. (**b**) Graphene on the flat polymer with an audio source. (**c**) Graphene on micropatterned polymer with an audio source [181]. Copyright 2023, ACS Appl. Mater. Interfaces.

Electrostatic loudspeakers generate sound waves through the vibration of a charged diaphragm sandwiched between two perforated metal electrodes, with core components including an air chamber, electrostatic actuation system, and diaphragm [180]. Designs utilizing suspended 2D material diaphragms (Figure 22b)—such as graphene and graphene oxide—leverage their atomic-scale thickness (0.3–2 nm), mechanical flexibility, and ultrahigh diameter-to-thickness ratios to achieve superior performance metrics, including broad dynamic range (DR), high sound pressure levels (SPL), and exceptional energy conversion efficiency.

Zhou et al. developed a full-range audio speaker employing a 4 mm-diameter, 60 μm-thick monolayer graphene diaphragm, demonstrating exceptional performance across the 20–20,000 Hz frequency spectrum [180]. The design not only achieved superior SPL output but also effectively suppressed sharp resonance phenomena in the 5–20 kHz range through graphene’s outstanding mechanical damping properties, with air damping enhancement promoting broadband acoustic response flattening. It is worth noting that the SiO_2_ insulation layer deposition process solves the short circuit problem of the diaphragm under high voltage driving, significantly improving the reliability of the device. In addition, Lee’s team engineered a bilayer graphene-polyimide (PI) composite diaphragm speaker (Figure 22a), demonstrating low-frequency sound generation (<1 kHz) in larger-diameter configurations [179]. Compared to pure graphene speakers, these graphene-transferred polymer film acoustic devices exhibit enhanced robustness, flexibility, and optical transparency while maintaining competitive electromechanical conversion efficiency—a critical advancement for flexible electronics and transparent audio-visual integrated systems.

However, these devices currently require high-voltage operation (200–1000 V) for electrostatic field modulation, posing electrical safety hazards and energy inefficiency concerns. Future advancements must prioritize optimizing pre-stress uniformity in 2D material diaphragms and developing robust interfacial encapsulation techniques to reconcile high-performance acoustic output with safe low-voltage operation [178,181,182,183,184,185,186,187,188,189,190].

##### Electrodynamic Speakers

**Figure 23:**
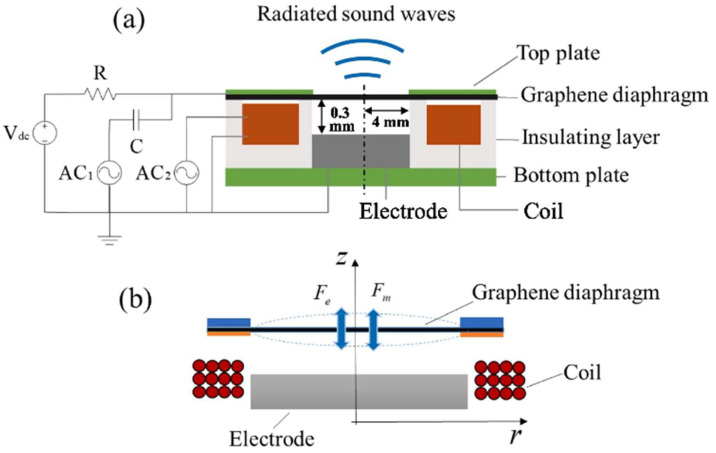
(**a**) Structure of electric graphene speaker based on suspended graphene diaphragm. A multilayer-graphene diaphragm is suspended between the top plate and the bottom electrode. The coil is embedded into the insulating layer. In the transmitting mode, the graphene diaphragm is biased by DC voltage V_dc_. The AC_1_ source is applied to the diaphragm (AC_1_ source is the AC signal from the channel 1 of the signal generator). (**b**) Mechanical schematic diagram of graphene diaphragm [193]. Copyright 2023, Ultrasonics.

Electrodynamic loudspeakers (dynamic coil loudspeakers) operate through electromagnetic induction principles, where a conductive coil embedded in the diaphragm vibrates under alternating-current-driven magnetic fields to generate sound waves [191]. The integration of graphene into such systems significantly enhances loudspeaker sensitivity, as demonstrated by Guo et al. [192], who developed electrodynamic graphene loudspeakers using suspended graphene membranes. Under identical operating conditions, these devices achieved nearly double the output sound pressure level (SPL) compared to coil-free electrostatic counterparts across nearly the entire audible frequency spectrum. Furthermore, Guo’s team proposed embedding conductive coils within insulating layers (Figure 23) to improve radiation performance in graphene-based acoustic transducers [193]. However, substantial Joule heating losses under high current densities result in energy efficiency markedly inferior to electrostatic approaches, highlighting a critical trade-off between electromechanical output and thermal management that requires innovative material engineering or hybrid actuation strategies to resolve.

##### Piezoelectric Speakers

Piezoelectric loudspeakers achieve direct electromechanical-acoustic transduction through the inverse piezoelectric effect, where alternating voltages induce periodic strain in piezoelectric membranes to generate sound—eliminating the need for DC biasing or coil structures while offering compact designs, customizable form factors, and superior high-frequency response [194,195]. However, their low-frequency performance has been fundamentally constrained by the thickness-dependent vibration modes of conventional bulk piezoelectric materials like lead zirconate titanate (PZT), which limit full-spectrum acoustic coverage. Although current research on independently employing 2D materials as piezoelectric loudspeaker diaphragms remains exploratory, their unique physical properties have demonstrated potential to revolutionize conventional piezoelectric acoustic devices [21].

##### Thermoacoustic Speakers

Thermoacoustic loudspeakers generate sound waves through periodic joule heating of ultrathin conductive membranes under alternating current, inducing thermal expansion oscillations in surrounding air molecules without requiring traditional vibrating diaphragms [196,197]. This technology offers three transformative advantages: (1) solid-state design eliminates mechanical constraints, enabling arbitrary curvilinear geometries and micron-scale devices with enhanced reliability; (2) ultrabroad frequency response spanning infrasound to ultrasound, facilitating applications in active noise cancellation and medical ultrasonography; and (3) safe low-voltage operation, circumventing the kilovolt-level risks of electrostatic systems [198].

**Figure 24:**
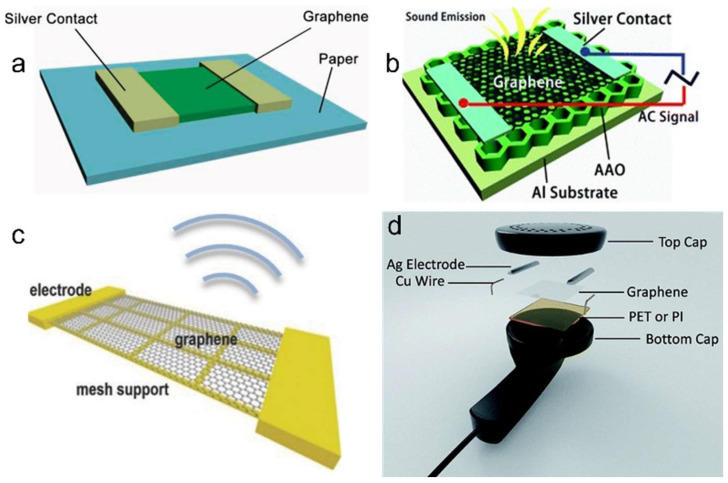
(**a**) Graphene-on-paper sound source devices. Structure of electric graphene speak. In this structure, a graphene film is located at the center of the paper. When sound frequency electric signal is applied to graphene through the silver contact, the joule heating will heat up the air near its surface, and then the periodicity of air vibration will form sound waves [199]. Copyright 2011, ACS Nano. (**b**) A schematic view of an AAO-based sound source device using SLG as the sound-emitting component. The working principle of SLG-SEDs can be described as when a sound frequency electric signal is applied to SLG, the joule heating will heat up the air near its surface, and then the periodicity vibration of the air will form sound waves [197]. Copyright 2012, Nanoscale. (**c**) The graphene-on-mesh thermoacoustic loudspeaker [196]. Copyright 2016, Small. (**d**) Schematic of the graphene earphone [197]. Copyright 2015, RSC Adv.

In 2011, Tian et al. demonstrated paper-supported suspended multilayer graphene as photothermal-acoustic conversion membranes (Figure 24a), achieving flat and broadband SPL output across audio and low ultrasonic frequencies [199]. Subsequent work in 2012 [197] optimized the architecture using anodic aluminum oxide (AAO) porous substrates to suspend monolayer graphene (Figure 24b), reducing the heater thickness and heat capacity per unit area (HCPUA), which significantly enhanced maximum SPL output.

Meanwhile, Suk et al. realized transparent and flexible thermoacoustic devices by hybridizing monolayer graphene with PDMS/PET composites [200]. Kim et al. integrated 28 nm-thick multilayer graphene onto PI mesh substrates and modulated the SPL output through controlled adjustments of the grid porosity (Figure 24c), establishing a proportional relationship between porosity variation and acoustic performance [201]. Tian et al. pioneered the first bendable graphene thermoacoustic earphones (Figure 24d) by strategically integrating graphene with PET and PI substrates, delivering smoother and louder SPL characteristics while maintaining mechanical flexibility [202].

The integration of 2D materials like graphene amplifies performance through their exceptional thermal conductivity and minimal heat capacity. However, challenges persist in low-frequency attenuation caused by thermal diffusion–air inertia mismatches and energy efficiency limitations due to parasitic heat conduction losses.

#### 4.3.2. Microphone

Microphones, serving as core devices for converting sound waves into electrical signals, are categorized into five primary types based on transduction principles: electromagnetic (including dynamic coil and ribbon types), capacitive, piezoelectric, piezoresistive (contact-type), and optical (fiber-optic and laser vibrometers) [198,199]. The first four types rely on electrical circuit designs—electromagnetic microphones generate current via coil motion in magnetic fields, capacitive microphones detect sound through diaphragm-backplate capacitance changes for high-precision measurement, piezoelectric microphones exploit material piezoelectric effects, and piezoresistive microphones gauge acoustic pressure via strain-induced resistance variations. In contrast, optical microphones transcend circuit limitations, with fiber-optic systems employing light phase modulation and laser vibrometers utilizing Doppler frequency shifts to achieve ultrahigh sensitivity and immunity to electromagnetic interference, making them indispensable for high-temperature industrial monitoring and MEMS device characterization. The integration of 2D materials further advances miniaturization and performance enhancement, and these innovations position 2D material-enhanced microphones at the forefront of next-generation acoustic technologies, spanning biomedical diagnostics, non-destructive testing, and quantum information systems.

Microphones are essentially a special pressure sensor. The exhibit exceptional performance across both the audible frequency range and ultrasonic regimes, with their technological evolution being profoundly enhanced by the unique attributes of suspended 2D materials [200,201]. Leveraging atomic-scale thickness and intrinsic flexibility, suspended 2D materials offer unparalleled advantages in acoustic sensing: their ultrahigh responsivity to minute pressure fluctuations and capacity to form ultrathin conductive diaphragms make them ideal platforms for next-generation microphone designs [202,203]. Over the past two decades, MEMS microphones have progressively replaced traditional systems in mobile devices through multi-unit integration technologies. Graphene-based capacitive microphones, employing atomic-scale thin layers and ultrahigh diameter-to-thickness ratios, overcome the inherent stiffness-frequency trade-off of conventional materials, achieving synergistic optimization of low stiffness, high resonant frequencies, broadband frequency response, and high sensitivity—attributes critical for smart devices integration [204].

**Figure 25:**
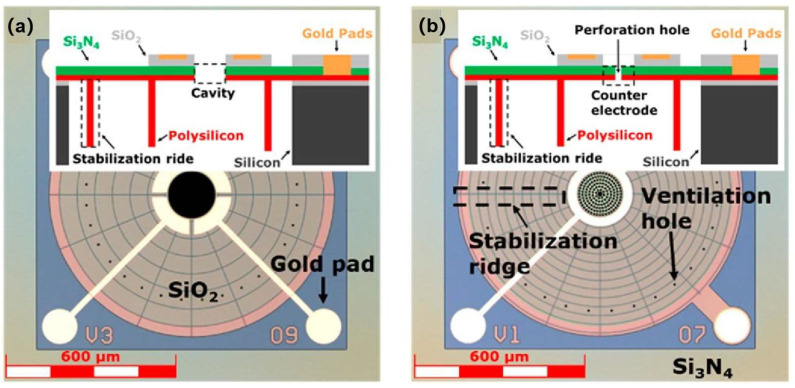
(**a**) A microphone (**b**) and a cross-sectional scanning electron micrograph (SEM) of a microphone structure, cut by focused ion beam (FIB) [205]. Si_3_N_4_, SiO_2_, gold pads, and polysilicon are colored green, grey, orange and red, respectively. The stabilization ridge is marked by the black dashed lines. Copyright 2019, ACS Appl. Nano Mater.

Graphene-based microphones (Figure 25) exhibit a remarkably low pull-in voltage below 2 V, enabling direct integration with low-voltage power systems in mobile devices like smartphones—a breakthrough resolving longstanding power compatibility challenges [205]. While current graphene microphone sensitivity remains at one-half to one-tenth of commercial MEMS counterparts, revolutionary structural designs achieve dimensional breakthroughs: graphene diaphragms demonstrate 17.5–27.5 times smaller diameters than silicon-based microphones. The incorporation of graphene not only enhances critical performance metrics but also drives device miniaturization, significantly reducing footprint while maintaining sensitivity and frequency response. This advancement facilitates cost-effective microphone production and expands applications in wearables (earbuds, smartwatches) and IoT systems, where space constraints and energy efficiency are paramount. The material’s mechanical robustness and atomic-scale thickness further enable novel acoustic architectures unattainable with conventional materials, creating opportunities for ultra-compact, high-fidelity audio solutions in next-generation electronics.

### 4.4. Photoelectric Device

Photodetectors play a pivotal role in high-speed information processing, low-power computing, highly sensitive sensing, and integrated optoelectronics. The primary drivers for their future development are miniaturization, ultra-low power consumption, ultra-high sensitivity and speed, and multifunctionality (e.g., polarization detection, self-powering). However, traditional substrate-supported structures are often limited by dielectric interference, interfacial defect scattering, and parasitic effects from the substrate, severely constraining the material’s intrinsic properties and ultimate device performance. Suspended 2D materials (e.g., graphene, MoS_2_, ReS_2_), with their unique substrate-free configuration, offer a key pathway to overcome these bottlenecks and achieve breakthrough optoelectronic performance. By eliminating substrate-induced scattering, loss, and charge traps, while providing an ideal platform for dual-side modulation and strain engineering, they exhibit ultra-high carrier mobility, low-loss light transmission, customizable photoresponse, and orders-of-magnitude improvements in responsivity and speed—precisely meeting the demands for next-generation high-performance, miniaturized, low-power optoelectronic devices.

#### 4.4.1. Logic Gates

**Figure 26:**
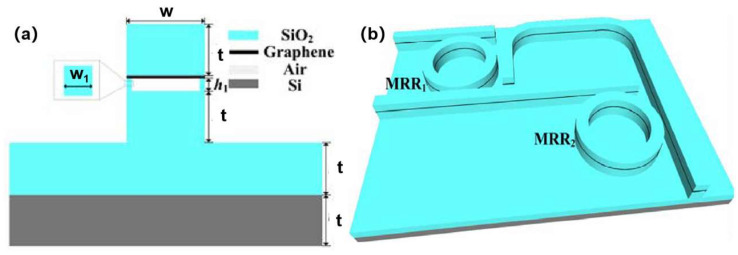
(**a**) Cross-sectional view of the proposed SGNRW. A SiO_2_–graphene–air–SiO_2_ structure is deposited on the SiO_2_/Si substrate. The width of the SiO_2_–graphene–air–SiO_2_ structure is labeled as w; the width of the air layer is (w−2w_1_); and the thicknesses of the air layer and the SiO_2_ layer are marked as h_1_ and t, respectively. For the SiO_2_/Si substrate, the thicknesses of the SiO_2_ layer and the Si layer are considered as t. (**b**) Three-dimensional schematic of the proposed plasmonic Feynman gate. The plasmonic Feynman gate comprises two cascaded MRRs, which are labeled as MRR_1_ and MRR_2_ [206]. Copyright 2019, IEEE Photonics J.

Suspended 2D materials, such as graphene, demonstrate unique advantages in Feynman gate applications for reversible photonic computing, achieving high extinction ratios and low-loss logic operations through tunable plasmonic effects and ultracompact designs. Suspended graphene nanoribbon waveguides (SGNRWs) serve as foundational platforms, where structural parameter optimization—including waveguide width and air layer thickness—enhances mode confinement and minimizes propagation losses [206,207]. Wang et al. proposed a suspended graphene nanoribbon waveguide (SGNRW) architecture featuring a unique SiO_2_–graphene–air–SiO_2_ multilayer structure (Figure 26) to implement plasmonic Feynman gates [206]. Compared to substrate-grounded plasmonic Feynman gates, the suspended configuration demonstrates superior performance in extinction ratio and crosstalk suppression.

The suspended architecture eliminates substrate dielectric interference, significantly improving the transmission efficiency of plasmonic modes. Future research may focus on multi-material integration (e.g., transition metal dichalcogenides), high-speed dynamic modulation, and fault-tolerant optimization to advance the practical implementation of photonic computing devices, potentially enabling breakthroughs in on-chip optical interconnects and energy-efficient neuromorphic computing systems.

#### 4.4.2. Field Effect Transistor Devices

**Figure 27:**
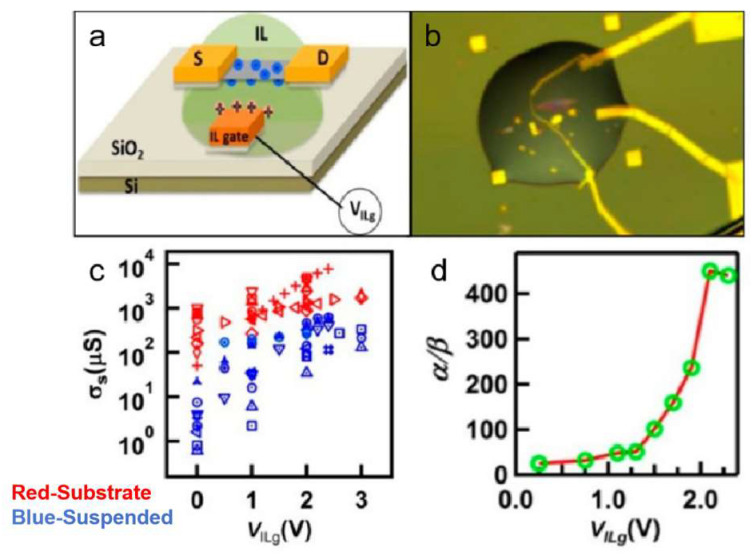
(**a**,**b**) Schematics and optical image of ionic liquid gating on a suspended device. Compared to as-fabricated devices, addition of IL invariably improves the device performance; even without applying any voltage to the counter electrode, the conductance typically increases by a factor of 10^3^ or 10^4^. (**c**) Zero-bias sheet conductance at different V_ILg_ of nine substrate-supported (blue) and nine suspended (red) samples. (**d**) Ratio of coupling efficiencies between IL gate and back gate as a function of V_Ilg_ [8]_._ Green circles are experimental values, and red line is guide to the eye. Copyright 2015, Nano Lett.

Suspended 2D materials enhance carrier control and electrical performance by eliminating substrate effects and minimizing interface defects, resulting in high carrier mobility and low parasitic capacitance that make them ideal for field-effect transistors (FETs). The suspended architecture enables the formation of electric double layers (EDLs) from ionic liquids (Figure 27) on both material surfaces, facilitating dual-side carrier injection that improves coupling efficiency and boosts conductivity by 1–2 orders of magnitude [8].

Wang et al. fabricated suspended MoS_2_ field-effect transistors employing an ionic liquid (IL) gating design that demonstrates exceptional charge modulation capability. By eliminating interface scattering effects inherent in conventional substrate-supported architectures, this suspended configuration significantly enhances carrier mobility. Compared to substrate-bound devices, the suspended structure achieves an IL gate coupling efficiency of 4.6 × 10^13^ cm^−2^V^−1^ alongside improved conductivity, enabling superior charge sensing performance and sustaining ultrahigh charge densities. Shin et al. fabricated suspended graphene FETs using a sandwich configuration, where the suspended active channel induces Dirac point shifting, reduced carrier density, and enhanced mobility due to the elimination of substrate-induced charge scattering and dielectric screening [208]. These advancements underscore the potential of suspended 2D materials in developing ultrahigh-performance electronic devices with tailored quantum transport properties.

#### 4.4.3. Photodetectors

Suspended 2D materials such as MoS_2_, ReS_2_, and graphene significantly enhance photodetector sensitivity, response speed, and functional diversity by eliminating substrate interference, modulating strain, and optimizing interfacial properties, demonstrating unique advantages in polarization detection, high-frequency operation, and low-power devices [209,210]. The suspended architecture enables pressure-gradient-induced strain engineering to precisely tailor materials’ electrical and optoelectronic characteristics—for instance, strain-induced symmetry breaking in MoS_2_ generates anisotropic optical responses for polarized light detection [211]. Suspended ReS_2_ devices exhibit suppressed interfacial traps (e.g., with SiO₂ substrates), achieving a carrier mobility of 8 cm^2^/V·s (versus 3 cm^2^/V·s in substrate-supported counterparts) and a record-fast response time of 20 μs, positioning them among the fastest TMDs-based photodetectors [212].

**Figure 28:**
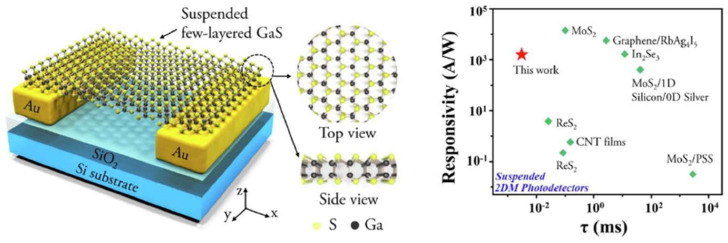
(**left**) Schematic diagram of suspended GaS photodetector and comparison of response time and response rate of different types of 2D-material-based photodetectors. The GaS and the metals were regarded as conductive channel, source, and drain electrodes, respectively. (**right**) Performance comparison of suspended 2D material photodetectors. Green makers are previous work, and red star represents the reference [213]. Copyright 2021, Mater. Des.

**Figure 29:**
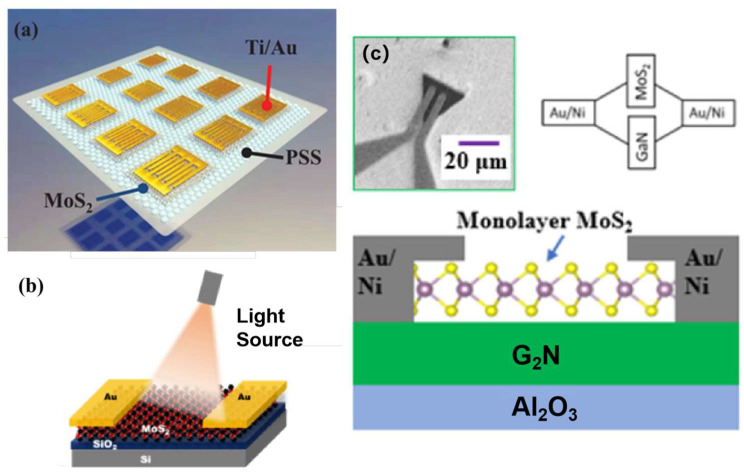
(**a**) Three-dimensional schematic view of the MoS_2_/PSS photodetector, in which the electrodes are Ti/Au [42]. Copyright 2021, Small. (**b**) Schematic of the device [214]. Copyright 2024, APL Energy. (**c**) The microscopic image (top left), charge transport equivalent circuit (top right), and the schematic of the fabricated detector (bottom) [215]. Copyright 2024, ACS Appl. Mater. Interfaces.

Zhong et al. further developed suspended GaS photodetectors (Figure 28) with ultrafast responses spanning the ultraviolet-to-visible spectral range, where the suspended structure mitigates interfacial scattering and surface defects, thereby unlocking GaS’s intrinsic merits and boosting device performance [213]. Prajapat et al. developed a suspended MoS_2_ photodetector (Figure 29b), achieving an ultrahigh responsivity of 1.02 × 10^4^ A/W, with a specific detectivity of 1.2 × 10^12^ Jones and a noise-equivalent power (NEP) as low as 1.56 × 10^−18^ W·Hz^−1/2^ [214]. In parallel, Vashishtha et al. engineered a novel self-powered bidirectional detector through MoS_2_/GaN heterojunction construction, demonstrating a peak responsivity of 631 mA·W^−1^ under zero bias for 365 nm ultraviolet light (Figure 29c) [215]. Liu et al. fabricated MoS_2_/PSS-based photodetectors that exhibited comprehensive superiority in photocurrent, response speed, and stability, highlighting the unique advantage of patterned substrates in localizing and enhancing short-wavelength photon interactions (Figure 29a) [42]. These advancements highlight suspended 2D materials’ exceptional optoelectronic tunability and efficiency gains, offering transformative prospects for next-generation photodetection technologies in quantum optoelectronics and on-chip integrated systems.

### 4.5. Cryo-Electron Microscopy

Cryo-EM has been widely utilized to resolve protein structures at atomic resolution. A critical challenge arises during standard cryo-EM sample preparation, where nearly all proteins predominantly adsorb at air–water interfaces, with interfacial interactions severely compromising reconstruction success and attainable resolution.

**Figure 30:**
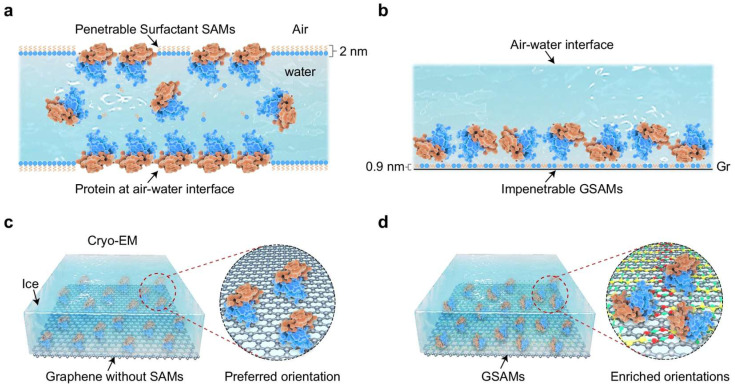
Schematic illustration showing that the surfactant SAMs at the air–water interface are easily penetrated by the protein particles, leading to the preferential orientation of particles. (**b**) Particles with various orientations adsorbed on the impenetrable GSAM superstructure, where surfactant SAMs lie flat on the graphene (Gr). The thickness of GSAMs (~0.9 nm) is thinner than that of the surfactant monolayer (~2 nm) standing upright in (**a**). (**c**,**d**) Schematic illustrations showing the protein particles adsorbed on the GSAM membrane with enriched orientations (**d**) compared to those on the pristine graphene surface (**c**). [52]. Copyright 2024, Nat. Commun.

Zhang et al. developed graphene liquid cells by π–π stacking two pristine single-crystal graphene membranes on TEM grids, eliminating polymer-induced contamination while maintaining atomic-level flatness across centimeter-scale suspended regions [96]. Furthermore, GSAMs (Figure 30) demonstrate exceptional capacity to host diverse proteins, providing precise control over protein–air–water interfacial interactions. GSAMs exhibit unique capabilities in enriching varied protein orientation distributions, enabling high-resolution structural determination of macromolecules spanning molecular weights from 700 to 52 kDa [52]. By combining graphene’s mechanical stability with SAM-enabled biochemical precision, this dual-functional interface resolves long-standing cryo-EM sample preparation challenges and establishes a universal platform for next-generation structural biology.

## 5. Discussion and Conclusions

In this review, we summarize recent advancements in the preparation, properties, and applications of suspended 2D materials. We also compare the advantages and disadvantages of suspended 2D materials versus substrate-supported ones and evaluate dry versus wet transfer methods. While significant progress has been made in the fabrication of suspended 2D materials in recent years, there remains a critical gap in developing manufacturing methods capable of producing large-scale, high-quality, and reproducible samples. The exceptional intrinsic properties of suspended 2D materials, combined with advances in their nanofabrication processes, hold substantial promise for transformative impacts in sensing and optoelectronic applications. In conclusion, future research on suspended two-dimensional materials will focus on overcoming fabrication limitations, with key priorities including advancing transfer methodologies to develop novel suspended 2D materials; improving heterojunction interface engineering (precisely regulating interlayer coupling, band alignment, and stress distribution); and developing advanced in situ characterization techniques to reveal intrinsic properties of suspended 2D materials. Building on these foundations, optimized design of next-generation high-performance electronic devices, ultra-sensitive sensors, NEMS, and quantum emitters will ultimately accelerate their practical deployment in information technology, sensing, and energy applications.

## Figures and Tables

**Table 1:** Comparison wet transfer and dry transfer.

	Fabrications	Yield	Challenges	Applications	References
Wet transfer	Two-step PMMA	>50%	Transfer integrity, PMMA residue removal	Most 2D materials	[48]
	PMMA as support layer (2-layer Gr)	2 × 3 µm^2^	Film flatness, interlayer/surface PMMA residue		[49]
	Inverted floating method	~50% for 200 μm suspended graphene	Device design, reagent flow control		[50]
	Cyclododecane as support layer	99% integrity (<10 μm)	Graphene-TEM grid adhesion, etchant residue		[51]
	Substrate-supported transfer	~99.5% coverage (GSAMs)	Graphene-TEM grid adhesion		[52]
Dry transfer	Mechanical exfoliation	Small-batch lab preparation	Large-area high-quality films		[29]
	Dry stamp transfer				[53]

**Table 2:** Comparison of the properties between supported 2D materials and suspended 2D materials.

	Graphene	Suspended Graphene	MoS_2_	Suspended MoS_2_	h-BN	Suspended h-BN
Mobility	2000–15,000 cm^2^/Vs [30]	250,000 cm^2^/Vs [30]	0.1 cm^2^/Vs [135]	0.9 cm^2^/Vs [135]	-	-
Thermal conductivity	600 W/mK [30]	2000–5000 W/mK [30]	-	34.5 ± 4 W/mK [136]	400 W/mK [33]	751 W/mK [33]
Young modulus		1 TPa [137]	0.27 GPa [138]	0.33 ± 0.07 Tpa [135]	-	0.865 ± 0.073 TPa [139]
